# FAST-GC: A Fully Adaptive Self-Tuning Ground Classification Algorithm for Multi-Platform LiDAR Sensors

**DOI:** 10.3390/s26217032

**Published:** 2026-11-06

**Authors:** Nadeem Fareed, Izaya Numata, Carlos Alberto Silva, Susan J. Prichard

**Affiliations:** 1 School of Forest, Fisheries, and Geomatics Sciences, University of Florida, Gainesville, FL 32611, USA; 2 Department of Geography, South Dakota State University, Brookings, SD 57006, USA; 3 School of Environmental and Forest Sciences, University of Washington, Seattle, Washington, WA 98195, USA; sprich@uw.edu

**Keywords:** LiDAR, ground-point classification, terrain modeling, sensor-adaptive processing, point-cloud processing, digital terrain model, ULS, TLS, ALS

## Abstract

**Highlights:**

**What are the main findings?**

• Development of a state-of-the-art multi-sensor ground-point classification (GPC) algorithm.
• A LiDAR multi-sensor and auto-parameterization GPC algorithm.
• Downstream derivative products—DEM, CHM, terrain, and canopy products.
• Deployable for various operating systems and cloud computing.

**What are the implications of the main findings?**

• Wall-to-wall massive LiDAR data processing.
• Derivative products for terrestrial ecosystems.
• Fully automated and efficient data processing capabilities.

**Abstract:**

Ground-point classification (GPC) is essential for light detection and ranging (LiDAR)-derived terrain and vegetation products, yet most of the well-established GPC algorithms often require parameter calibration across varying terrain, vegetation, point densities, and sensor modalities—airborne laser scanning (ALS), unoccupied aerial vehicle laser scanning (ULS), mobile laser scanning (MLS), and terrestrial laser scanning (TLS). This study introduces FAST-GC, a fully adaptive, self-tuning, parameter-free GPC algorithm designed for ALS, ULS, MLS, and TLS LiDAR sensors. FAST-GC was evaluated against cloth simulation filtering (CSF), progressive TIN densification (PTD), and progressive morphological filtering (PMF) across benchmark sites (n = 6), with additional terrain-complexity, slope, and sampling-density assessments. FAST-GC maintained high ground completeness with limited commission errors, particularly under complex topography. At slopes of 70–80°, the FAST-GC-derived digital terrain model (DTM) mean absolute error (MAE) was 1.00 m, compared with 3.12–12.75 m for the reference counterparts—CSF, PMF, and PTD. Across 20 ALS sites, reducing median point density from 9.84 to 3.84 points/m^2^ maintained F1 ≈ 0.97, intersection over union (IoU) ≈ 0.95, and Matthews correlation coefficient (MCC) ≈ 0.85, while DTM MAE increased slightly from 0.07 to 0.11 m. FAST-GC-derived DTM (0.5 m) from concurrent ALS, ULS, and TLS showed strong cross-platform agreement (R^2^ and concordance correlation coefficient (CCC) ≥ 0.99; RMSE = 0.10–0.16 m). Processing 382 ALS tiles (~23 km^2^) further demonstrated scalable execution and automated terrain-product generation. These findings establish FAST-GC as a reproducible, sensor-aware framework that preserves terrain morphology across heterogeneous LiDAR datasets without scene-specific parameter calibration.

## 1. Introduction

The use of airborne laser scanning (ALS) and its sister technological niche—unpiloted aerial system laser scanning (ULS), mobile laser scanning (MLS), and terrestrial laser scanning (TLS)—has significantly increased in recent years [1,2,3,4]. Laser scanners, whether deployed independently or concurrently in a multi-platform conundrum across aerial and terrestrial platforms, now provide unprecedented three-dimensional (3D) information on terrestrial ecosystems, enabling advanced investigations in forest ecosystems [5,6], forest management [7], wildfire dynamics [8,9], terrain characterization [10], forest ecology [11], hydrology [12], and geomorphology [13,14].

To generate key LiDAR-derived products—DTM, digital elevation models (DEMs), canopy height models (CHMs), and Individual Tree Detection (ITD)—numerous parametric, machine learning (ML), and deep learning (DL) approaches have been investigated [15,16,17,18,19]. Among these approaches, ground-point classification (GPC)—also termed bare-earth extraction—is a fundamental LiDAR data processing step that isolates topography from off-terrain (OT) natural and man-made objects, such as vegetation and buildings [20,21,22,23]. The increasing availability of national-scale LiDAR datasets is enabling scientific analyses at spatial resolutions and extents that were previously unprecedented [24]. Due to the availability of higher sampling densities—Aggregate Nominal Point Density (ANPD)—greater than 2 pts./m^2^, derived products (DEM, CHMs) with a resolution of 0.5 m have been developed [25]. Modern laser scanners—ALS, ULS, MLS, and TLS—provide much higher ANPDs to generate high-resolution (HR) DEMs with cm level resolution [14]. However, generating HR-DEMs at centimeter-scale spatial resolutions requires accurate GPC, which constitutes a critical processing step for producing HR downstream products, such as CHM and topographic derivatives, capable of capturing fine-scale terrain variability for applications in forestry and terrestrial ecosystem investigations [26,27].

### 1.1. Ground Classification Algorithms

Over the past three decades, several GPC algorithms have emerged and are widely implemented in software environments such as Point Data Abstraction Library (PDAL)—Python programming and lidR–R programming [10,28,29]. GPC algorithms can be broadly categorized into distinct algorithmic families based on their underlying principles. Morphological filters, e.g., progressive morphological filter (PMF) [30] and simple morphological filter (SMRF), rely on structuring elements and elevation thresholding to separate ground from non-ground points. Surface-based and interpolation approaches, such as progressive TIN densification (PTD), iteratively construct and refine a terrain surface using geometric constraints. Curvature-based methods, notably Multiscale Curvature Classification (MCC) [13], exploit local surface curvature and scale-space analysis to distinguish terrain features. Physical simulation approaches, particularly the cloth simulation filter (CSF)-based GPC, use a mass–spring system that simulates a cloth draping over an inverted surface [31,32]. Additionally, slope- and elevation-threshold methods, such as modified slope-based filters (MSBFs) [33] and extended local minimum (ELM) techniques [34]—apply heuristic rules based on local gradients [21].

Despite the methodological advances, GPC algorithm families are fundamentally built on assumptions of terrain continuity, elevation gradients, and neighborhood relationships [22,23,35,36]. Consequently, most approaches require explicit parameterization, including window size, slope thresholds, iteration limits, or stiffness coefficients, e.g., CSF [20,37,38]. A fundamental challenge is parameter sensitivity to topographic complexities, where optimal performance depends heavily on site-specific tuning, thereby reducing transferability across diverse forest ecosystems and topographic complexities [39,40,41]. Morphological, slope-based, and TIN-based ground filters remain sensitive to terrain complexity and initialization, as steep gradients, abrupt discontinuities, and errors in seed selection can propagate through the GPC process, whereas algorithms with several user-defined adaptive parameters require parameter tuning to maintain good performance across heterogeneous landscapes [32,42]. Nevertheless, most GPC algorithms were originally developed and tested using ALS, which typically exhibits relatively uniform sampling and moderate ANPD. When applied to cross-platform LiDAR modalities, including ULS, MLS, and TLS, these methods encounter additional challenges closely associated with sensor modalities rather than topographic regime [20,43,44,45]. These include heterogeneous point density, anisotropic sampling geometry, occlusion effects, and complex vertical structures [46,47]. To account for both topographic regime and sensor-related sensitivities, the parameter adjustment becomes significantly challenging when a ground truth or explicit accuracy validation is absent. Under such conditions, GPC algorithms are being used based on published resources where studies investigate the sensitivity of these parameters, along with providing optimized parameters through extensive trial-and-error analysis [22,47].

### 1.2. Contributions

To address these challenges, this study introduces FAST-GC, a fully automated, self-tuning GPC algorithm designed to reduce the dependence on scene-specific parameter calibration while preserving terrain morphology across heterogeneous topographic complexities and sensor modalities [17]. The fundamental research gap addressed in this study is the persistent dependency of existing GPC algorithms on user-defined parameters and site-specific optimization, which limits their adaptability and transferability across heterogeneous terrain, vegetation conditions, point densities, and LiDAR acquisition platforms [20,39,40]. These limitations become particularly pronounced in complex topographic environments, where inadequate parameterization can lead to the omission of legitimate ground points [48], terrain contamination with off-terrain (OT) objects—particularly near-surface vegetation, and reduced fidelity of the reconstructed DEM/DTM despite availability of sufficient ground points [49]. Unlike approaches that depend primarily on predefined spatial scales or externally optimized classification thresholds [50], FAST-GC derives its terrain-support characteristics from the statistical distribution of point clouds, followed by unsupervised candidate ground points in inverted 3D space with complementary ground-recovery and hierarchical filtering operations addressing sensor modalities and terrain complexity without user-specific parameterization and calibration. This study objectively targets four interconnected objectives:

(i) To develop a data-driven sensor-mode (ALS, ULS, TLS) adaptive GPC algorithm capable of accommodating variations in terrain complexity, vegetation 3D vertical characterization, and point densities without site-specific parameter optimization—a widely recognized limitation of existing GPC algorithms [50].
(ii) To quantify GPC accuracy and terrain-surface reconstruction fidelity relative to widely used GPC algorithms (CSF, PMF, PTD), particularly under steep terrain and reduced ground-point availability [47].
(iii) To examine the consistency of independently reconstructed terrain surfaces across ALS, ULS, and TLS acquisition geometries—an algorithm that is insensitive to sensor modalities and carrier platforms [36,48].
(iv) To assess the operational applicability of the framework for large-area, tile-based LiDAR processing and subsequent terrain-product generation by jointly considering point-wise classification performance, terrain-surface accuracy (DEM), sampling-density sensitivity, cross-platform consistency, and processing stability [51].

## 2. Materials and Methods

### 2.1. Workflow

**Figure 1:**
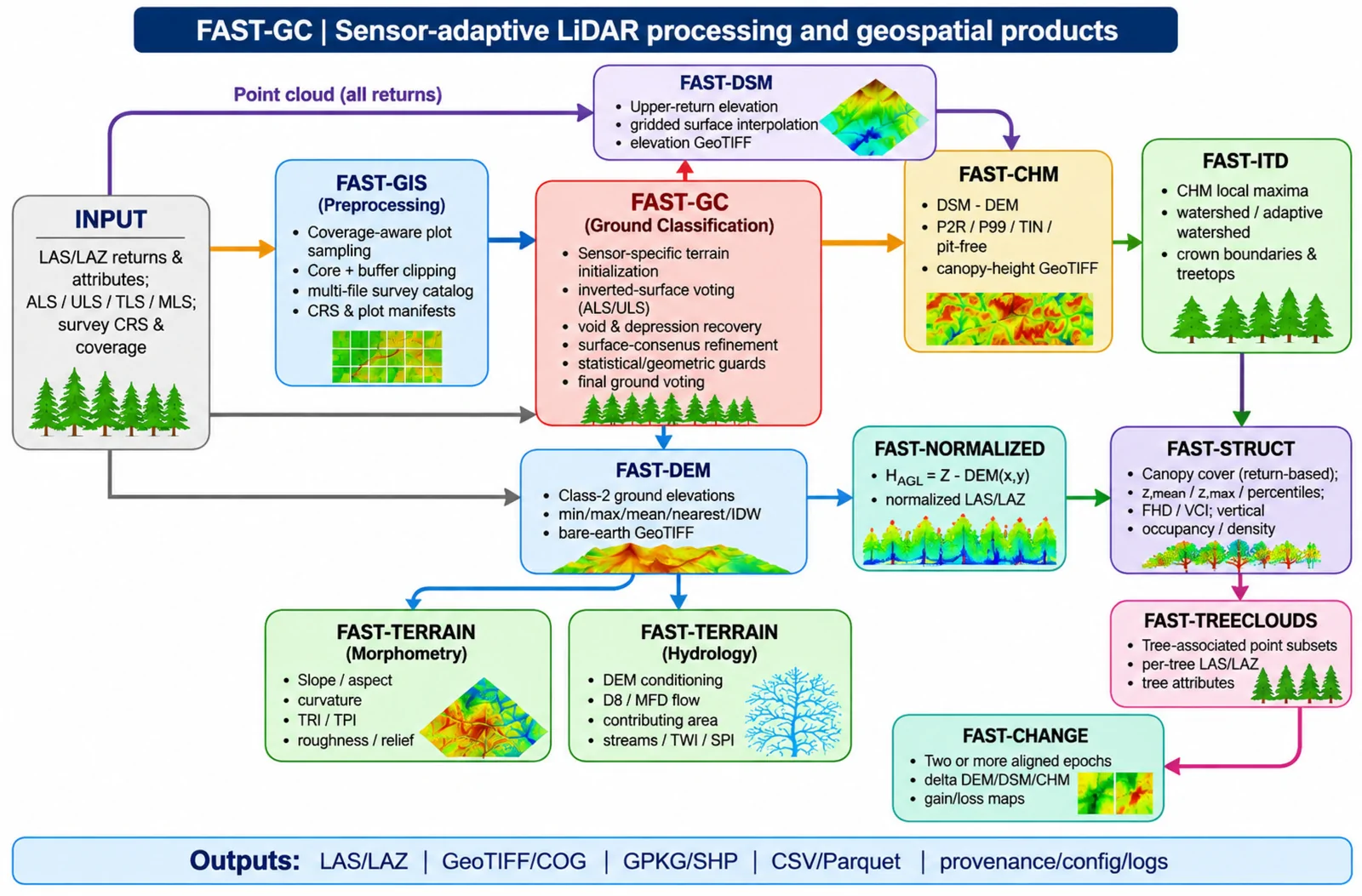
Integrated architecture of FAST-GC for sensor-adaptive LiDAR ground classification and geospatial product generation. The workflow combines survey-aware spatial preprocessing, sensor-specific terrain classification, surface and height-model generation, geomorphometric and hydrological analysis, forest structural characterization, individual-tree detection, and multitemporal change assessment. Products are generated from LAS/LAZ inputs through reproducible, configurable processing workflows.

To the best of our knowledge, FAST-GC is the first parameter-free GPC algorithm that introduces a sensor-aware, data-adaptive GPC pipeline, in which terrain-support criteria are dynamically determined based on the LiDAR acquisition platform and the spatial sampling characteristics of the input point clouds, enabling ground classification across heterogeneous sensor configurations and terrain conditions. FAST-GC provides a modular workflow for LiDAR data processing (Figure 1). The available modules include the following: (a) input LAS/LAZ data cataloging, user-defined tiling, and tile-manifest generation; (b) sensor-aware FAST-GC ground classification for ALS, ULS, and TLS point clouds, followed by DEM/DTM generation and point-cloud height normalization; (c) spike-free digital surface model (DSM) generation (Khosravipour et al., 2016) [52] and merging of tile-wise outputs; (d) terrain and topographic derivatives, including slope, aspect, hillshade, curvature, terrain convergence index (TCI), and topographic wetness index (TWI); (e) canopy height model (CHM) generation using point-to-raster (p2r) [53], 99th-percentile (p99), pit-free CHM (Khosravipour et al., 2014) [54], percentile, percentile-top, and percentile-band approaches; (f) ITD using Watershed (Vincent and Soille, 1991) [55] and (Yun et al., 2021) methods [56]; (g) forest structural metrics, including foliage height diversity (FHD) (MacArthur and Horn, 1969) ([57], vertical complexity index (VCI) [4], canopy cover (CC), standard deviation of height [z (SD)], number of points [Points (n)], and mean height (z mean); and (h) DEM of Difference (DoD) change detection (Wheaton et al., 2010) [58].

The modular workflow illustrated in Figure 1 provides an overview of the broader processing capabilities of the FAST-GC Python package. The software is publicly available through the Python Package Index (PyPI; https://pypi.org/project/fastgc/; accessed on 22 October 2026) for installation and through GitHub (https://github.com/nadeemfareed/FAST-GC; accessed on 22 October 2026) for source-code access and version tracking. Comprehensive technical documentation, including descriptions of all FAST-GC products, their scientific validation and application domains, processing configurations, reproducible workflows, practical examples, and computational performance assessments, is provided in the accompanying FAST-GC Book (https://nadeemfareed.github.io/fastgcBOOK/; accessed on 22 October 2026). The following subsections focus specifically on the development and evaluation of the FAST-GC ground classification algorithm.

### 2.2. Tile-Enabled Fully Automated Workflow

FAST-GC is designed for large-scale LiDAR processing by partitioning input point clouds, provided either as individual files or collections of files, into user-defined tiles with spatial buffers. The buffers preserve neighboring-point support across tile boundaries and reduce edge artifacts during seamless merging of tiles to produce spatially continuous products (e.g., classified and normalized ground points, CHM, DSM, and DEM) in wide-area capacity [59]. Although spatial tiling is widely used in existing Python and R programming LiDAR packages, e.g., lidR [10], FAST-GC couples tiling with a persistent JavaScript Object Notation (JSON) manifest [60] that records the processing configuration, input file sources, sensor mode, spatial extent, tile geometry, buffer size, point counts, point density (pts./m^2^), spatial occupancy, and support statistics required to reconstruct and manage the automated FAST-GC processing pipeline. Dataset-level summaries of tile sampling characteristics are additionally derived and used by FAST-GC to adapt selected internal processing scales when local sampling support deviates substantially from the overall dataset, thereby accommodating variations associated with sensor sampling and spatial heterogeneity. An additional safeguard is applied to incomplete terminal tiles generated along dataset boundaries. Small edge fragments and geometrically narrow sliver tiles are identified during tile planning and automatically consolidated with adjacent spatial tiles before the point-cloud tiles are written—a unique feature that is available in FAST-GC to avoid any data loss during tile processing.

### 2.3. Algorithm Design and Mathematical Background

FAST-GC is conceptually motivated by CSF in the inverted point-cloud 3D domain [61,62]. In CSF, the point clouds are inverted using point elevation (z), and a simulated cloth is allowed to conform to the inverted surface [62]. FAST-GC retains inversion as a useful geometric transformation but replaces cloth simulation with deterministic reconstruction of an observed terrain-support manifold—a thin membrane of topographic surface by separating it from off-the-terrain objects (OT). FAST-GC accomplishes this using three tiers of information directly from point clouds to operate without user-defined parameterization over complex topographic regimes: (i) input sensor mode, which defines the nature of the sensor platform, is followed by (ii) statistical information directly obtained from tiles and (iii) automatic derivation of the operational parameter initiation in 3D inverted space to deal with point densities, occlusion, and height range in each tile to facilitate statistical inference in inverted 3D space.

FAST-GC begins with sensor-specific baseline configurations for ALS, ULS, and TLS but adapts the spatial support scale to the sampling characteristics of individual input point-cloud tiles. Let *s_d_* denote the dataset-level scale selected from the observed support-density regime. A bounded tile-level adjustment, Δ*s_t_*, is subsequently derived from the relative local support of the tile, including the median point count and occupancy of 2 m grid cells and, where required, point density. The effective support scale, *s_t_*, is

(1) *s_t_* = clip (*s_d_*+Δ*s_t_*, *s*_min_, *s*_max_)

where *s*_min_ and *s*_max_ are sensor-specific bounds. Thus, sparsely supported tiles receive a modest increase in spatial support, whereas exceptionally dense tiles permit a smaller support scale. Selected spatial support parameters are then adapted from their sensor-specific baseline values (Equation (2)).

(2) $$h=\mathrm{clip}\left(h_{0},s_{t},\;h_{\min,}h_{\max}\right),n_{\min}=\mathrm{round}\left[\mathrm{clip}\left(\frac{n_{0}}{\sqrt{s_{t}}},n_{L},n_{U}\right)\right]$$

where *h*_0_ and *n*_0_ are the baseline voting-cell size and minimum number of supporting neighboring cells, respectively; *h* is the adapted horizontal cell size; and *n_L_* and *n_U_* define the allowable sensor-specific support range. Candidate-cell size and other spatial support distances are adapted analogously within bounded ranges. This procedure changes the spatial scale of evidence collection without altering the underlying classification logic. Let the input point cloud be

(3) $$P={\left\{p_{i}=\left(x_{i},y_{i},z_{i}\right)\right\}}_{i=1}^{N}$$

where *x_i_* and *y_i_* denote horizontal coordinates and *z_i_* is the original elevation of return *i*. FAST-GC constructs the initial terrain support in an inverted vertical space, such that low-elevation returns in the original point cloud become upper-envelope observations after inversion—an identical geometric transformation implemented in the CSF algorithm [62]. Using a reference elevation *z*_ref_, the inverted elevation of point *i* can be expressed as

(4) $${\tilde{z}}_{i}=z_{\mathrm{ref}}-z_{i}$$

where ${\tilde{z}}_{i}$ denotes the inverted elevation. Because this transformation reverses vertical ordering, a point with the lowest original elevation *z_i_* has the highest inverted elevation ${\tilde{z}}_{i}$, which can be presented as

(5) $$\arg\;\max_{i}\;{\tilde{z}}_{i}=\arg\;\min_{i}\;z_{i}$$

The horizontal domain is partitioned into cells of width *h*. Within each occupied cell *c*, FAST-GC selects the point having the maximum elevation as the initial terrain candidate—theoretically, a candidate point in the given cell with the highest probability of being a legitimate ground point,

(6) $$q_{c}^{\left(0\right)}=\;{\tilde{z}}_{i},{\tilde{L}}_{c}^{\left(0\right)}={\tilde{z}}_{q_{c}^{\left(0\right)}}$$

where $q_{c}^{\left(0\right)}$ is the index of the selected candidate points and ${\tilde{L}}_{c}^{\left(0\right)}$ is its elevation in inverted space. To reduce dependence on the arbitrary position of the grid origin, candidate selection is repeated using a second grid displaced by one-half of the cell width along both horizontal axes, as presented as:

(7) $$\left(x_{0}^{\prime },y_{0}^{\prime }\right)=\left(x_{0},+\frac{h}{2}y_{0}+\frac{h}{2}\right)$$

This produces a second set of inverted-space candidates, ${\tilde{L}}_{c}^{\left(1/2\right)}$. After mapping the offset-grid candidates to the base grid, the two candidate layers are combined by retaining the upper support envelope in inverted space.

(8) $${\tilde{L}}_{c}=\max\left({\tilde{L}}_{c}^{\left(0\right)},{\tilde{L}}_{c}^{\left(1/2\right)}\right)$$

For cells in which both candidate points are available from both layers, the point with the highest elevation is retained, whereas the available point is used when only one grid contributes support, given that one of the layers contains empty grid cells. The half-cell displacement provides an alternative spatial partition for returns located near base-grid boundaries and thereby reduces grid-origin sensitivity and compensates for the empty grid cells originating from the base layer. The combined provisional support is subsequently anchored to their original locations as candidate ground seeds in unclassified point clouds. For each base-grid cell *c*, FAST-GC selects the points whose inverted elevation is closest to the provisional support ${\tilde{L}}_{c}$:

(9) $$q_{c}^{*}=\underset{i\in c}{\arg\;\min}\left|{\tilde{z}}_{i}-{\tilde{L}}_{c}\right|,\;\;{\tilde{S}}_{c}={\tilde{z}}_{q_{c}^{*}}$$

where $q_{c}^{*}$ is the index of the selected return and ${\tilde{S}}_{c}$ is its snapped elevation in inverted space. This snapping operation constrains the support layer to elevations represented by actual LiDAR returns before terrain voting. FAST-GC next evaluates whether each snapped support ${\tilde{S}}_{c}$ is consistent with its surrounding terrain. Let 𝒩*_c_*(*R*) denote the square neighborhood surrounding cell *c*, where *R* is the half-width of the square voting neighborhood, expressed in grid cells.

(10) $${\tilde{m}}_{c}={\mathrm{median}}_{j\in N_{c}\left(R\right)}{\tilde{S}}_{j},{\tilde{d}}_{c}=\max\left[{\mathrm{median}}_{j\in N_{c}\left(R\right)},|{\tilde{S}}_{j}-{\tilde{m}}_{c}|d_{\min}\right]$$

where ${\tilde{m}}_{c}$ represents the local center of the inverted terrain-support elevations, ${\tilde{d}}_{c}$ is their unscaled median absolute deviation (MAD), and *d*_min_ prevents the robust dispersion estimate from collapsing in locally uniform terrain adopted from [63]. A neighboring support ${\tilde{S}}_{j}$ contributes to the terrain vote when

(11) $$|{\tilde{S}}_{j}-{\tilde{m}}_{c}|\leq k_{\mathrm{MAD}}\left(1.4826\right){\tilde{d}}_{c}$$

where *k*_MAD_ controls the allowable standardized deviation and 1.4826 provides the conventional Gaussian-consistent scaling of MAD [63]. If at least *n*_min_ neighboring support cells satisfy Equation (11), the voted terrain-support elevation in inverted space at cell *c* is estimated as

(12) $${\tilde{T}}_{c}=\mathrm{median}\left\{{\tilde{S}}_{j},:j\in N_{c}\left(R\right)|{\tilde{S}}_{j}-{\tilde{m}}_{c}|\leq k_{\mathrm{MAD}}\left(1.4826\right){\tilde{d}}_{c}\right\}$$

Grid cells lacking a valid terrain estimate after neighborhood voting are iteratively filled from neighboring finite terrain estimates using a 3 × 3 median neighborhood, after which NaN-aware separable smoothing is applied to obtain the continuous inverted terrain membrane. The resulting membrane is then transformed back to the original elevation space as:

(13) $$\hat{T}\left(x,y\right)=z_{\mathrm{ref}}-\tilde{T}\left(x,y\right)$$

Here, $\hat{T}\left(x,y\right)$ is the reconstructed terrain membrane in the original coordinate system [62]. Importantly, the voting stage suppresses isolated low or high observations before surface completion, thereby reducing the influence of vegetation returns and other vertically inconsistent support. The reconstructed membrane is bilinearly sampled at the horizontal position of each return, and its signed vertical residual is defined as:

(14) $$r_{i}=z_{i}-\hat{T}\left(x_{i},y_{i}\right)$$

where positive residuals represent returns above the estimated terrain and negative residuals represent returns below it. Rather than applying a globally fixed elevation threshold, FAST-GC adjusts the admissible residual according to local terrain geometry:

(15) *τ_i_* = clip(*τ*_0_, +, *k_s_* *g_i_* + *b_i_*, *τ*_min,_*τ*_max_)

where *τ*_0_ is the baseline vertical tolerance, *k_s_* is the slope-adaptation coefficient, $g_{i}=∥\nabla \hat{T}\left(x_{i},y_{i}\right)∥$ is the local terrain-gradient magnitude, and *b_i_* is a bounded allowance associated with abrupt local terrain transitions. The latter is derived from local breakline relief.

(16) $$\rho_{c}=|{\hat{T}}_{c}-{\mathrm{median}}_{3\times 3}{\left(\hat{T}\right)}_{c}|,b_{i}=\mathrm{clip}\left(1.5,\rho_{i},0,\;00.25\right)$$

where *ρ_i_* is the bilinearly sampled relief at point *i*. Unlike curvature, *ρ_i_* retains units of elevation and represents local departures from the surrounding terrain surface at crests, concavities, and slope transitions. Initial ground membership is then assigned using an asymmetric residual interval.

(17) $$G_{i}^{\left(0\right)}=\left\{\begin{array}{cc} 1, & -2\tau_{i}\leq r_{i}\leq \tau_{i},\\ 0, & \mathrm{otherwise}, \end{array}\right.$$

where $G_{i}^{\left(0\right)}=1$ denotes initial ground membership. The asymmetric interval permits greater downward deviation from the reconstructed terrain membrane than upward deviation while maintaining a stricter constraint against above-terrain returns. The initial terrain classification is subsequently refined using a sequence of spatial-consistency tests designed to address terrain voids, depressions, locally weak ground support, canopy leakage, isolated ground labels, and tile-edge inconsistencies. These operations use the already-reconstructed terrain membrane and neighboring point evidence rather than introducing an independent classification model. Recovery is, therefore, restricted to points that exhibit sufficient local terrain support, while contamination guards remove ground labels that are vertically or spatially inconsistent with the surrounding terrain manifold. Detailed formulations and parameter bounds for these refinement operations are provided in the Supplementary Materials (Note S1).

### 2.4. Benchmark and Cross-Validation Datasets

For GPC quantitative evaluation, the principal benchmark was assembled from the ALS dataset of Jin et al. (2020), acquired in the southern Sierra Nevada Mountains, California (CA), USA [17]. The original dataset comprised twenty 500 × 500 m sites distributed among four study areas: Providence, San Joaquin Range, Courtwright Road, and Wolverton–Tokopah. The region encompasses substantial topographic variation and mixed-conifer forest dominated by white fir (*Abies concolor*), ponderosa pine (*Pinus ponderosa*), and incense cedar (*Calocedrus decurrens*), with black oak (*Quercus kelloggii*) and canyon live oak (*Quercus chrysolepis*). Across the twenty original sites, mean canopy cover ranged from 0 (non-forested) to 90%, mean tree height from 0 to 25.9 m, mean elevation (m.a.s.l.) from 426 to 3347 m, and mean terrain slope from 6.0° to 41.5° [17].

The ALS data were acquired in August 2010 using an Optech GEMINI Airborne Laser Terrain Mapper (ALTM) mounted on a twin-engine Piper PA-31 Chieftain. The system was operated at a pulse repetition rate of 100 kHz and a scanning frequency of 50 Hz, with a ±14° scan angle and a single-swath width of 233.26 m. Adjacent swaths overlapped by more than 50%, and the sensor recorded up to four echoes per emitted pulse. The mean point density across the acquisition was approximately 10.27 pts m^−2^. Independent vertical assessment was conducted by the data provider using 243 road checkpoints collected with a vehicle-mounted GPS; the reported root mean square error (RMSE) of LiDAR elevations relative to these checkpoints was 0.024 m and was described as spatially homogeneous among the study sites [17].

**Figure 2:**
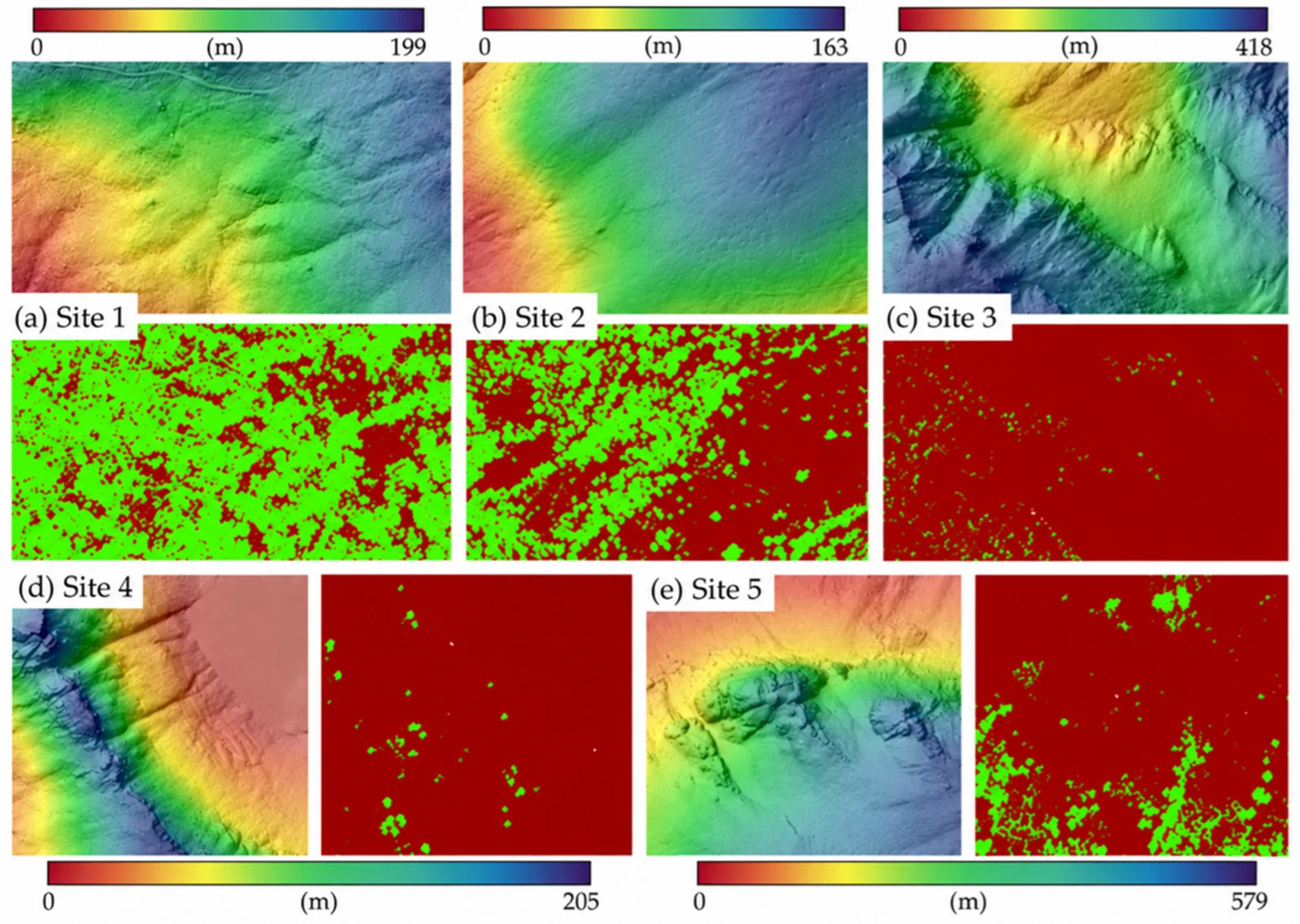
Topographic and reference classification characteristics of five ALS benchmark sites: (**a**) Site 1; (**b**) Site 2; (**c**) Site 3; (**d**) Site 4; and (**e**) Site 5. Elevation-shaded terrain surfaces illustrate topographic morphology, while corresponding reference classifications distinguish ground (red) and non-ground (green) points. The sites represent contrasting vegetation and terrain complexity.

A subset (n = 5) was assembled from Jin et al. (2020) [17] for the controlled benchmarking following a similar approach over the same geographic region [42]. To avoid confusion between the numbering used here and that of the source dataset, the selected blocks were renumbered as FAST-GC benchmark Sites 1–5 (Table 1). Benchmark Site 1 combines source blocks 6 and 8 of Jin et al. (2020) [17], whereas benchmark Sites 2–5 use source blocks 1, 12, 13, and 15, respectively (Table 1). Hereafter, all site numbers refer exclusively to the FAST-GC benchmark numbering unless explicitly stated otherwise. The subset was selected to represent contrasting sources of ground classification difficulty and canopy cover conditions (see Figure 2).

Site 1 represents the vegetation-dominated end of the benchmark (Figure 2a). Its two combined forest blocks have mean canopy cover of 72% and 54%, mean tree heights of 24.9 and 13.7 m, and mean terrain slopes of 15.8° and 12.4°, respectively. In the combined benchmark point cloud, only 40.36% of points are reference ground, providing a strong test of ground discrimination where non-ground returns are abundant. Site 2 represents an intermediate vegetation–terrain condition, with 30% mean canopy cover, 10.3 m mean tree height, 17.1° mean slope, and 69.09% reference ground (Figure 2b).

The dominant filtering challenge changes substantially at Sites 3 and 4. These sites contain 96.86% and 98.26% reference ground, respectively, while mean canopy cover decreases to 17% and 1% (Figure 2c,d). Their terrain, however, is considerably more demanding: mean slopes are 36.1° and 28.2°, with maximum slopes reported by Jin et al. (2020) [17] of 88.3° and 87.1°, respectively. These sites, therefore, provide particularly useful tests of whether a GPC algorithm can retain legitimate ground across steep and rapidly varying terrain with minimal vegetation. Site 5 combines both sources of complexity. It has 29% mean canopy cover and 41.5° mean terrain slope—the highest mean slope among the selected benchmark sites—while the distributed point cloud spans approximately 579 m in elevation within 0.25 km^2^ (Figure 2e). The controlled benchmark (n = 5) consequently encompasses vegetation-dominated, intermediate, exposed steep-terrain, and combined vegetation–extreme-terrain conditions rather than relying solely on differences in ground/non-ground class proportions.

Reference-label quality was an additional reason for selecting the Jin et al. (2020) [17] benchmark. The authors first generated preliminary ground classifications using TerraScan and subsequently inspected the classified point clouds manually using cross-sectional visualization in LiDAR360 [17]. Erroneously classified points were corrected, and additional legitimate ground points were recovered during this procedure. For the six source blocks used in the present study, the reported proportions of manually corrected points were 10.99%, 13.18%, 14.44%, 25.44%, 0.12%, and 33.61% for source blocks 1, 6, 8, 12, 13, and 15, respectively. Additionally, three complementary datasets were used to examine aspects of FAST-GC performance that could not be assessed from the Jin et al. (2020) [17] benchmark alone.

First, selected scenes from the OpenGF benchmark (Sites 6–7, Table 1) were used as external stress tests under environmental conditions distinct from the controlled benchmark [64]. OpenGF is a large-scale ALS ground-filtering dataset containing more than 542 million labeled points over more than 47 km^2^ and representing nine terrain-scene types across four countries. An urban scene was included because built environments provide comparatively well-defined exposed surfaces for examining classification behavior in a setting outside the primary design domain of FAST-GC, which is unbuilt terrestrial landscapes (Site 6, Table 1). Mountainous forest scenes were additionally used to evaluate performance under complex terrain and sparse ground-returning conditions. Quality-control inspection of these scenes (Site 7), including profile/transect-based examination, indicated that the supplied ground labels could represent a comparatively conservative delineation by only incorporating, as reported by the authors, “high-quality ground labels”. Consequently, OpenGF was retained as an operational external reference rather than treated as equivalent to the manually corrected Jin et al. (2020) [17] benchmark. Performance was, therefore, examined in two complementary ways: (i) conventional point-wise comparison of FAST-GC, PMF, CSF, and PTD against the high-quality OpenGF ground labels and (ii) pairwise comparison among the independently generated GPCs to determine whether apparent disagreement with the high-quality OpenGF ground labels was also reflected in disagreement among the four GPC algorithms.

Second, a multi-platform LiDAR dataset from the central European forest of Weiser et al., 2022a was used to evaluate cross-platform consistency among independently acquired ALS, ULS, and TLS modalities [49,65]. The dataset used comprises three plots, BR01, BR07, and SP02 in Bretten and Karlsruhe, Baden-Württemberg, Germany, with spatially overlapping acquisitions from ALS, ULS, and TLS sensors. Since the GPC point clouds were not generated specifically as manually validated ground-filtering references, these datasets were used primarily to assess whether FAST-GC could reconstruct spatially consistent HR-DEM (0.5 m) under substantially different acquisition geometries and point densities rather than for conventional point-wise accuracy benchmarking. The original acquisition characteristics of the ALS, ULS, and TLS datasets across the three temperate mixed-forest plots are summarized in Table 2, including sensor configurations, acquisition conditions, point densities, and spatial coverage.

**Table 1:** LiDAR datasets used to evaluate FAST-GC across contrasting acquisition platforms, terrain, vegetation, and urban conditions. Study area identifies the test site; application specifies its evaluation role; location gives geographic origin; collection denotes LiDAR platform; area (km^2^) indicates spatial coverage; (pts m^−2^) denotes mean point density; GC/N-GC (%) gives reference ground/non-ground proportions; elevation range (m) indicates minimum–maximum elevations; and canopy conditions summarize vegetation and terrain characteristics. Datasets represent diverse rather than balanced landscape categories; Site 8 detailed survey characteristics are provided in Table 2.

Study Area	Application	Location	Collection	Area (km^2^)	(pts m^−2^)	GC/N-GC (%)	Elevation Range (m)	Canopy Conditions
Site 1	Benchmark	San Joaquin Range, CA, USA	ALS [17]	0.50	11.82	40.36/59.64	1822.27–2020.75	Dense forest; substantial canopy structure and locally steep terrain.
Site 2		Courtwright Road, CA, USA		0.25	10.30	69.09/30.91	2590.04–2752.96	Moderately forested terrain; balanced vegetation–terrain challenge.
Site 3		Wolverton–Tokopah, CA, USA		0.25	8.49	96.86/3.14	2800.35–3217.89	Sparse vegetation with steep, strongly varying topography.
Site 4				0.25	8.30	98.26/1.74	2904.96–3109.68	Coupled vegetation and complex/steep terrain challenge.
Site 5				0.25	9.05	78.24/21.76	2175.60–2754.79	Extreme relief and very steep terrain with moderate vegetation.
Site 6	Cross-validation/Urban	Arnhem, Netherlands	ALS [64]	0.50	18.57	42.17/57.83	7.20–38.76	Urban site with substantial trees, natural areas, and plantations
Site 7	Cross-validation	USA, New Zealand	ALS [4]	0.75	16.16	14.28/85.72	83–883	Mountain sites
Site 8	Cross-platform validation	Germany	ALS/ULS/TLS [49]	—	—	—	—	Temperate mixed forest
Site 9	Stability test	Lubrecht, USA	ALS [66]	23	20.07	—	1516–2019	Mountainous mixed-conifer forest

**Table 2:** LiDAR acquisition characteristics for three temperate mixed-forest plots used to evaluate FAST-GC across airborne laser scanning (ALS), UAV laser scanning (ULS), and terrestrial laser scanning (TLS) [49]. Plot identifies the study site; forest type describes vegetation conditions; collection denotes the LiDAR platform; sensor identifies the commercial scanning instrument; leaf indicates leaf-on or leaf-off conditions; acquisition date specifies the survey date; area (ha) represents the original LAS/LAZ XY bounding-box area; density (pts m^−2^) denotes point density calculated from this area; points (M) indicates the total number of points in millions; elevation range (m) gives minimum–maximum point elevations; and flight AGL (m) indicates approximate flight altitude above ground level. The symbol ≈ denotes estimated values, and—indicates not applicable.

Plot	Forest Type	Collection	Sensor	Leaf	Survey Date	Area ^1^ (ha)	(pts m^−2^)	Points (M)	Elevation Range (m)	AGL ^2^ (m)
BR01	Temperate mixed forest	ALS	RIEGL VQ-780i	On	05 Jul 2019	16.21	77.3	12.53	236.5–336.3	≈691.6
		ULS	RIEGL miniVUX-1UAV	On	12 Sep 2019	24.91	158.3	39.42	204.5–411.6	≈67.4
		ULS	RIEGL miniVUX-1UAV	Off	04 Dec 2019	15.88	328.6	52.18	235.5–340.9	≈61.6
		TLS	RIEGL VZ-400	On	03 Jul 2019	26.25	1333.2	349.90	235.1–320.4	—
BR07	Temperate mixed forest	ALS	RIEGL VQ-780i	On	05 Jul 2019	3.14	101.7	3.20	275.8–353.8	≈669.0
		ULS	RIEGL miniVUX-1UAV	On	24 Aug 2019	31.72	147.6	46.83	180.6–363.5	≈66.8
		ULS	RIEGL miniVUX-1UAV	Off	26 Mar 2020	30.43	187.8	57.16	209.6–375.2	≈71.3
		TLS	RIEGL VZ-400	On	19 Jul 2019	22.57	1369.0	308.97	271.8–354.3	—
SP02	Temperate mixed forest	ALS	RIEGL VQ-780i	On	05 Jul 2019	3.94	71.4	2.81	270.7–326.3	≈675.3
		ULS	RIEGL miniVUX-1UAV	On	03 Sep 2019	16.48	186.9	30.81	195.8–345.0	≈62.6
		ULS	RIEGL miniVUX-1UAV	Off	01 Apr 2020	21.83	297.0	64.82	228.6–335.9	≈62.0
		TLS	RIEGL VZ-400	On	23 Aug 2019	12.32	2506.6	308.75	274.2–358.9	—

Notes: ^1^ Area represents the LAS/LAZ XY bounding box; density is calculated as total points per unit area. TLS density is not directly comparable to airborne density. ^2^ AGL = above ground level; approximate values (≈) were derived from flight trajectories and FAST-GC terrain elevations. For nominal flight heights, consult the original survey publication. — = not applicable. Exact AGL and LiDAR survey-related information can be found at [49].

Third, a spatially extensive ALS dataset from the Lubrecht Experimental Forest, Montana, USA, was used to assess the landscape-scale processing stability of FAST-GC and the spatial consistency of the resulting CHM at 0.5 m resolution (Site 9). The original survey was acquired by Quantum Spatial for the University of Montana during 27–30 June 2015 using a Leica ALS70 sensor operated at approximately 1400 m above ground level. The complete acquisition encompassed 61,237 acres and achieved an average first-return density of 20.07 pts m^−2^ [66]. The Lubrecht Experimental Forest represents a topographically heterogeneous mixed-conifer landscape in western Montana, providing extensive variation in terrain, canopy cover, and ground-return availability [66,67,68]. For the present study, an approximately 23 km^2^ subset of the available ALS coverage was used as a large-area evaluation dataset. Unlike the smaller datasets used for point-wise accuracy assessment, this dataset was intended to examine whether FAST-GC could be applied systematically at landscape scale while maintaining spatially coherent terrain reconstruction.

### 2.5. Evaluation Approaches

To benchmark the performance of FAST-GC, a comprehensive review of the existing literature was conducted to identify widely used and well-established GPC algorithms [64,69]. The selection was guided by three key criteria. First, the algorithms are implemented in open-source environments, ensuring accessibility and reproducibility for the broader research community [10,47,70]. Second, the selected methods represent distinct and widely recognized GPC algorithm families, enabling a balanced and meaningful comparison [29]. Based on these criteria, the performance of FAST-GC was evaluated against three representative approaches, CSF, PMF, and PTD, all of which are implemented and available in the lidR package [10]. Finally, all three above-mentioned GPC algorithms have been extensively applied and validated in prior studies to classify point clouds from ALS, ULS, and TLS, as evidenced by their frequent use in the published literature, e.g., CSF, PMF, and PTD, and, therefore, qualified for comparison [29,64,71,72,73].

Point-based GPC performance was evaluated using binary reference labels, with ground as the positive class and non-ground as the negative class. Performance was quantified using intersection over union (IoU), precision, recall, F1-score, Matthews correlation coefficient (MCC), omission error (OE), and commission error (CE) (Equations (18)–(24)). IoU was calculated for the ground class. Precision and recall quantify the purity and recovery of predicted ground points, respectively, while F1-score summarizes their balance. MCC incorporates all four components of the binary confusion matrix and provides a complementary measure of classification agreement under class imbalance. OE represents the proportion of reference ground points omitted from the predicted ground class, whereas CE represents the proportion of predicted ground points labeled as non-ground in the reference. Here, TP, FP, TN, and FN denote true-positive, false-positive, true-negative, and false-negative points, respectively [74].

(18) IoU = TP/(TP + FP + FN)

(19) Precision = TP/(TP + FP)

(20) Recall = TP/(TP + FN)

(21) F_1_ = (2 × Precision × Recall)/(Precision + Recall) = 2TP/(2TP + FP + FN)

(22) MCC = (TP·TN − FP·FN)/√[(TP + FP)(TP + FN)(TN + FP)(TN + FN)]

(23) OE = [FN/(TP + FN)] × 100 = (1 − Recall) × 100

(24) CE = [FP/(TP + FP)] × 100 = (1 − Precision) × 100

For the ALS benchmark sites (n = 6), the supplied ground/non-ground labels were used as the principal reference for quantitative evaluation. FAST-GC, PMF, CSF, and PTD predictions were compared point by point against the same reference labels using the metrics defined above. This evaluation constituted the principal-controlled comparison of ground classification performance and was used to examine algorithm behavior across the represented forest and terrain conditions.

The OpenGF evaluation was treated separately because it served as an external benchmark and stress test rather than as an extension of the principal-controlled ALS evaluation. All four algorithms were first evaluated against the supplied OpenGF ground/non-ground labels using the same point-wise metrics, thereby preserving direct comparison with the published benchmark. The supplied labels were, therefore, retained unchanged as the external reference, but the resulting reference-based metrics were interpreted together with a second, reference-independent analysis. Pairwise positive agreement among FAST-GC, PMF, CSF, and PTD classifications was calculated to determine the extent to which independently applied algorithms identified a common ground-point population. This complementary analysis was not used to establish ground-truth correctness; rather, it provided a means of distinguishing disagreement specific to the supplied reference from broader disagreement among the four GPC algorithms.

Because point-wise classification performance does not directly quantify the geometric fidelity of the reconstructed terrain, terrain-surface performance was evaluated independently using HR-DEM at 0.5 m resolution. For *n* spatially corresponding DEM cells, the elevation residual was defined as *e_i_* = *z_i_*_,pred_ − *z_i_*_,ref_. Reference-based DEM accuracy was quantified using bias, mean absolute error (MAE), RMSE, and the 95th percentile of absolute error (*P*_95_) (Equations (25)–(28)). Bias represents systematic vertical displacement, MAE represents the magnitude of absolute vertical error, RMSE gives greater weight to larger deviations, and *P*_95_ characterizes the upper tail of the absolute-error distribution [73].

For cross-platform terrain-surface comparisons, normalized bias (nBias), coefficient of determination (*R*^2^), concordance correlation coefficient (CCC), and normalized median absolute deviation (NMAD) were additionally calculated (Equations (29)–(32)) [75,76].

(25) Bias = (1/n) Σ_i=1_^n^ e_i_

(26) MAE = (1/n) Σ_i=1_^n^ |e_i_|

(27) RMSE = √[(1/n) Σ_i=1_^n^ e_i_^2^]

(28) P_95_ = Q_0.95_(|e_i_|)

(29) $$\mathrm{nBias}\;\left(\%\right)=100\times \mathrm{Bias}/\bar{z}\mathrm{ref}$$

(30) R^2^ = 1 − [Σ_i=1_^n^(y_i_ − ŷ_i_)^2^/Σ_i=1_^n^(y_i_ − ȳ)^2^]

(31) $$\mathrm{CCC}=2\mathrm{Cov}\left(x,y\right)/[\mathrm{Var}\left(x\right)+\mathrm{Var}\left(y\right)+{\left(\bar{x}-\bar{y}\right)}^{2}]$$

(32) NMAD = 1.4826 × median(|e_i_ − median(e_i_)|)

These metrics characterize relative vertical bias, linear correspondence, concordance, and robust residual dispersion, respectively. Cross-platform statistics were interpreted as measures of terrain-surface consistency rather than absolute accuracy because the independently acquired ALS, ULS, and TLS datasets do not provide a common point-wise benchmark or HR-DEMs [49].

For comparison, the GPC algorithms were applied using a single fixed parameterization across all benchmark sites: PTD (th1 = 1.2, th2 = 0.1, tol = 10, max_edge = c(0, 1.5), and n = 8), PMF (window sizes = 3, 6, 9, and 12 m; corresponding elevation thresholds = 0.10, 0.57, 1.03, and 1.50 m), and CSF (slope smoothing = TRUE, classification threshold = 1.0 m, cloth resolution = 1.0 m, rigidness = 1, maximum iterations = 500, and time step = 1.0) [73,77]. These configurations were selected from documented ALS implementations and literature-supported operating ranges rather than optimized separately for individual sites [10]. Because the benchmark encompasses contrasting conditions ranging from dense canopy conditions (Sites 1–2) and moderate-terrain environments to steep and strongly varying topography (Sites 3–5), the same parameterization was retained across all sites to provide a reproducible common operating configuration.

## 3. Results

### 3.1. ALS Ground Classification Benchmark

To quantify the GPC performance on Jin et al. (2020) [17] controlled benchmark sites (n = 5), point-wise classification performance was evaluated (Table 3, Figure 2) using identical operational settings for all GPC algorithms (250 m processing tiles/chunks with a 5 m buffer). FAST-GC was compared with CSF, PMF, and PTD. Table 3 reports precision, recall, F1-score, intersection over union (IoU), Matthews correlation coefficient (MCC), commission error (CE) %, and omission error (OE) %.

Across the ALS benchmark sites (n = 5), FAST-GC achieved the highest overall point-wise GPC performance, with macro-average recall, F1-score, IoU, and MCC of 0.96, 0.98, 0.96, and 0.81, respectively (Table 3). Precision remained (≈1.00) for all algorithms, indicating that differences among algorithms were driven primarily by their ability to retain benchmark ground points rather than by excessive inclusion of non-ground points as ground. FAST-GC maintained a macro-average CE of 0.35%, while OE was 3.36%. Among the GPC algorithms, CSF was the strongest overall competitor, with macro-average F1-score, IoU, and MCC of 0.92, 0.86, and 0.63, respectively, followed by PMF and PTD. PTD produced the lowest CE (0.06%) but also the highest OE (23.12%), indicating a comparatively conservative GPC that minimized FPs at the cost of substantial ground omissions [18,31].

The performance pattern differed systematically with terrain and vegetation setting. Sites 1 and 2 represent the highly forested conditions (Table 1; Figure 2a,b) but differ in ground-point returns. Site 1 contained 59.64% non-ground and dense canopy structure over locally steep terrain, whereas Site 2 comprised a larger ground fraction (69.09%), deciphering the topographic complexities in detail. Nonetheless, CSF and FAST-GC showed comparable performance. At Site 1, CSF achieved F1, IoU, and MCC values of 0.99, 0.97, and 0.98, respectively, compared with 0.98, 0.96, and 0.96 for FAST-GC. At Site 2, CSF and FAST-GC achieved F1 ≈ 1.00 and IoU ≈ 0.99, with PMF also maintaining similar performance. In contrast, PTD recall was 0.82 at Sites 1 and 2, corresponding to OE values of 18.46% and 17.96%, respectively. These results indicate that forest sites with substantial canopy returns, both FAST-GC and CSF, maintained effective ground recovery; on the contrary, substantially higher OE for PTD reflected a more conservative GPC response [31,78].

The relative GPC performance changed substantially at Sites 3 and 4, where ground returns constituted 96.86% and 98.26% of the point clouds, respectively, and the main classification challenge was associated with steep and strongly varying terrain rather than dense canopy conditions (Table 1; Figure 2c,d). FAST-GC maintained the highest overall performance under strongly varying terrain, with recall of 0.96 and 0.97 and F1-scores of 0.98 and 0.99 at Sites 3 and 4, respectively, while limiting OE to 3.81% and 2.89%. CSF was the closest reference method at both sites, but its recall declined to 0.79 and 0.85, corresponding to substantially higher OE values of 20.99% and 14.73%. PTD and PMF were similarly omission-prone; at Site 4, both achieved a recall of 0.75 with approximately 25% OE, while at Site 3, PMF showed the lowest ground retention (recall = 0.72; OE = 28.32%). These results are consistent with the sensitivity of conventional filtering approaches to steep slopes and rapidly varying terrain. Progressive TIN densification can perform well in mountainous terrain but depends strongly on the quality of initial terrain seeds and parameter thresholds [31], while CSF can provide a high-quality initial terrain yet may misclassify points during refinement in steep mountainous areas. Forest-focused comparisons have likewise reported smoothing or omission of steep terrain by PMF and PTD and increased vegetation-related commission by CSF under complex conditions [40,78].

**Figure 3:**
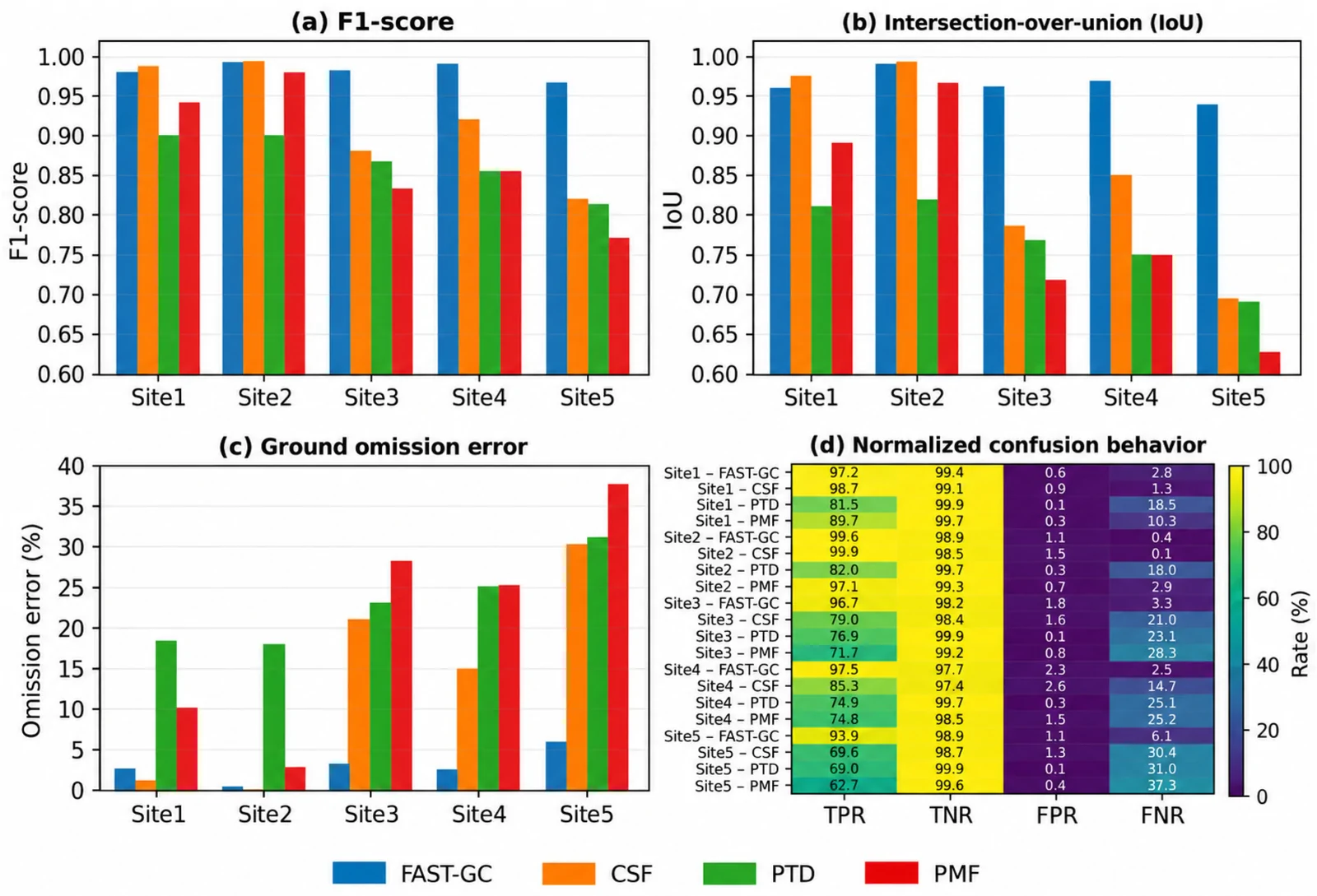
Site-wise point-level ground classification performance of FAST-GC, CSF, PTD, and PMF across the five controlled ALS benchmark sites. (**a**) F1-score, (**b**) intersection over union (IoU), (**c**) ground omission error (OE), and (**d**) normalized confusion behavior expressed as true-positive rate (TPR), true-negative rate (TNR), false-positive rate (FPR), and false-negative rate (FNR). Panels (**a**–**c**) illustrate changes in classification agreement and ground retention among sites, while panel (**d**) resolves the corresponding balance between correct ground retention and non-ground rejection.

Site 5 represents the combined challenge of substantial vegetation and extreme terrain relief, with 21.76% non-ground returns and an elevation range of approximately 579 m (2175.60–2754.79 m; Table 1; Figure 2e). FAST-GC maintained the highest overall performance, with recall of 0.93, F1-score of 0.96, IoU of 0.92, and MCC of 0.85, while limiting OE to 6.82%. CSF and PTD showed nearly identical F1-score and IoU values (0.82 and 0.69, respectively), indicating similar deterioration in ground retention, whereas PMF showed the greatest ground loss, with recall of 0.63, F1-score of 0.77, and OE of 37.35% [79]. The substantially higher OE of the GPC algorithms under the combined terrain–vegetation effect indicates increasing difficulty in retaining ground returns as surface complexity increases. This pattern is consistent with previous forest benchmarking studies reporting that morphological approaches can omit substantial ground in structurally complex environments and that a GPC algorithm’s performance depends strongly on terrain form, canopy conditions, and parameter optimization [79]. A visual comparison of ALS benchmark sites (n = 5) shows the clear trend of quantitative evaluation of all GPC algorithms with a changing terrain-vegetation regime (Figure 3).

The quantitative error distribution in Figure 3 further supports the divergence among GPC algorithms as terrain complexity increased. Across Sites 3–5, FAST-GC maintained high ground retention, with true-positive rate (TPR) ranging from 93.2% to 97.1% and false-positive rate (FPR) from 2.89% to 6.82% while maintaining low CE (0.04–0.33%). In contrast, the reference algorithms generally retained very low CE but showed pronounced reductions in TPR, indicating that their declining F1-score and IoU were driven primarily by omission of benchmark ground points rather than increased acceptance of non-ground points. This behavior was most evident at Site 5, where OE increased to 30.38%, 31.00%, and 37.35% for CSF, PTD, and PMF, respectively, compared with only 6.82% for FAST-GC. The very low CE of PTD and PMF, therefore, reflects, in part, a more conservative approach characterized by higher ground OE rather than uniformly improved ground/non-ground discrimination [40,79,80]. Nevertheless, Table 3 and Figure 3 show that differences among GPC algorithms became more pronounced as terrain complexity increased. Whereas FAST-GC and CSF performed comparably under dense canopy conditions (Sites 1–2), FAST-GC maintained substantially higher ground retention across the steep and topographically complex areas (Sites 3–5), with substantially lower OE than the counterparts—CSF, PMF, and PTD [79].

### 3.2. Urban Test Site

An additional ALS site (n = 1) from the OpenGF (Site 6) was used to examine the transferability of FAST-GC to an urban environment (area ≈ 0.50 km^2^) outside its primary application domain of natural and forested landscapes. The site is characterized by relatively gentle terrain interspersed with buildings, vegetation, and other constructed surface features, introducing ground/non-ground discrimination challenges that differ from those encountered in the controlled forest benchmark (n = 5)—see Figure 3 and Table 3.

CSF achieved the highest overall classification agreement, with an F1-score of 0.95, IoU of 0.91, and MCC of 0.92 (Table 4). FAST-GC achieved an F1-score of 0.92, IoU of 0.86, and MCC of 0.87, outperforming PTD (F1 = 0.89; IoU = 0.81; MCC = 0.83) and PMF (F1 = 0.87; IoU = 0.78; MCC = 0.78) on these metrics. FAST-GC retained nearly all reference ground points (recall = 0.99; OE = 0.27%), demonstrating strong ground preservation despite its lower precision (0.86) and elevated CE (13.17%), which indicate the inclusion of some non-ground points in urban areas [29].

PTD exhibited the highest precision (0.95) and lowest CE (4.72%), representing improvements of 0.09 and 8.45 percentage points, respectively, over FAST-GC. However, this reduction in commission was accompanied by substantially lower recall (0.84) and greater OE (15.92%), indicating a trade-off between non-ground rejection and reference-ground preservation. FAST-GC maintained F1, IoU, and MCC higher than PTD, reflecting its more favorable overall classification agreement despite greater CE. PMF achieved nearly complete ground retention but exhibited the highest CE (22.45%), whereas CSF provided the most favorable overall balance between ground retention and non-ground rejection for this urban scene.

**Figure 4:**
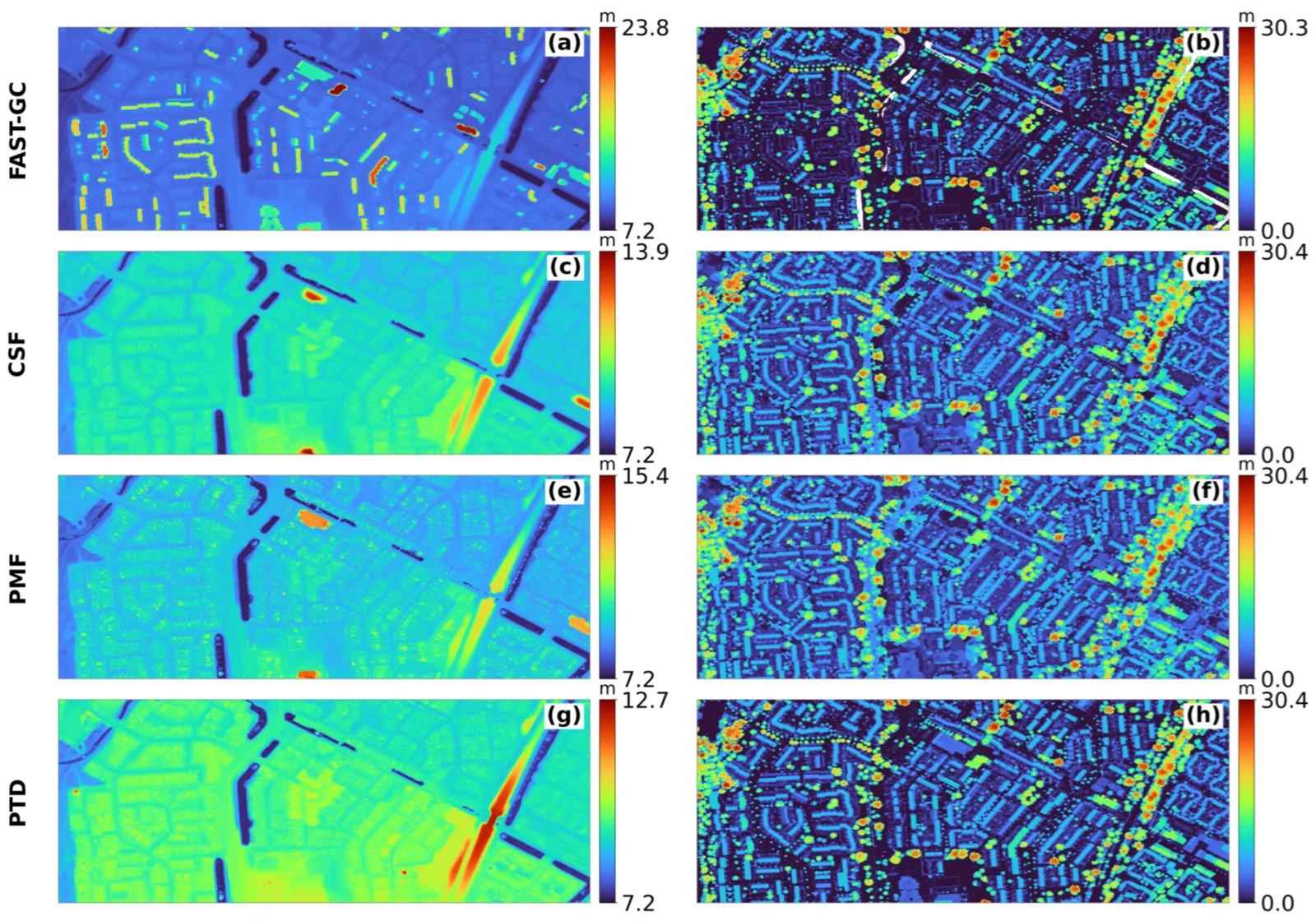
Comparison of DEMs (left column) and CHMs (right column) derived from ALS data for urban Site 8 using four ground classification methods: (**a**,**b**) FAST-GC, (**c**,**d**) CSF, (**e**,**f**) PMF, and (**g**,**h**) PTD. All DEMs and CHMs were generated at 0.5 m spatial resolution. Color bars indicate elevation (DEM) and canopy height (CHM) in meters, with limits corresponding to the actual minimum and maximum values of each raster.

Qualitative assessment of DEMs and CHMs further demonstrates the differences in urban ground point retention among the GPC algorithms (Figure 4). FAST-GC retained several prominent building structures in its DEM, consistent with its elevated CE (13.17%), given its in-built tolerance for abrupt terrain transitions (Figure 3), although a substantial portion of the buildings was correctly classified as non-ground (Figure 4a). In contrast, CSF and PMF removed most of the building structures but exhibited more spatially extensive near-surface retention of non-ground across built-up areas (Figure 4c,e), while PTD produced a smoother terrain surface with fewer visible building-related features (Figure 4g). Notably, PMF exhibited a higher CE (22.45%) than FAST-GC despite suppressing more buildings than FAST-GC, suggesting that near-surface non-ground features may contribute substantially to CE (Table 4, Figure 4e). CSF showed similar near-surface irregularities but achieved a lower overall CE (9.21%) than FAST-GC and PMF (Table 4). Nevertheless, FAST-GC maintained comparatively consistent elevations across surrounding ground surfaces, indicating more effective suppression of these visible near-surface irregularities compared to other GPC algorithms (Figure 4a,c,e,g). Despite differences in built-up retention in DEMs, all GPC algorithms preserved recognizable urban tree canopy distributions in their corresponding CHMs (Figure 4b,d,f,h), with FAST-GC exhibiting relatively consistent low-height regions comparable to PTD (Figure 4b,h). Collectively, these observations highlight differences between building-related and near-surface residual features and suggest that FAST-GC retains useful urban canopy information despite its susceptibility to only building-related contamination while maintaining more consistent bare-earth points.

### 3.3. Qualitative Analysis

**Figure 5:**
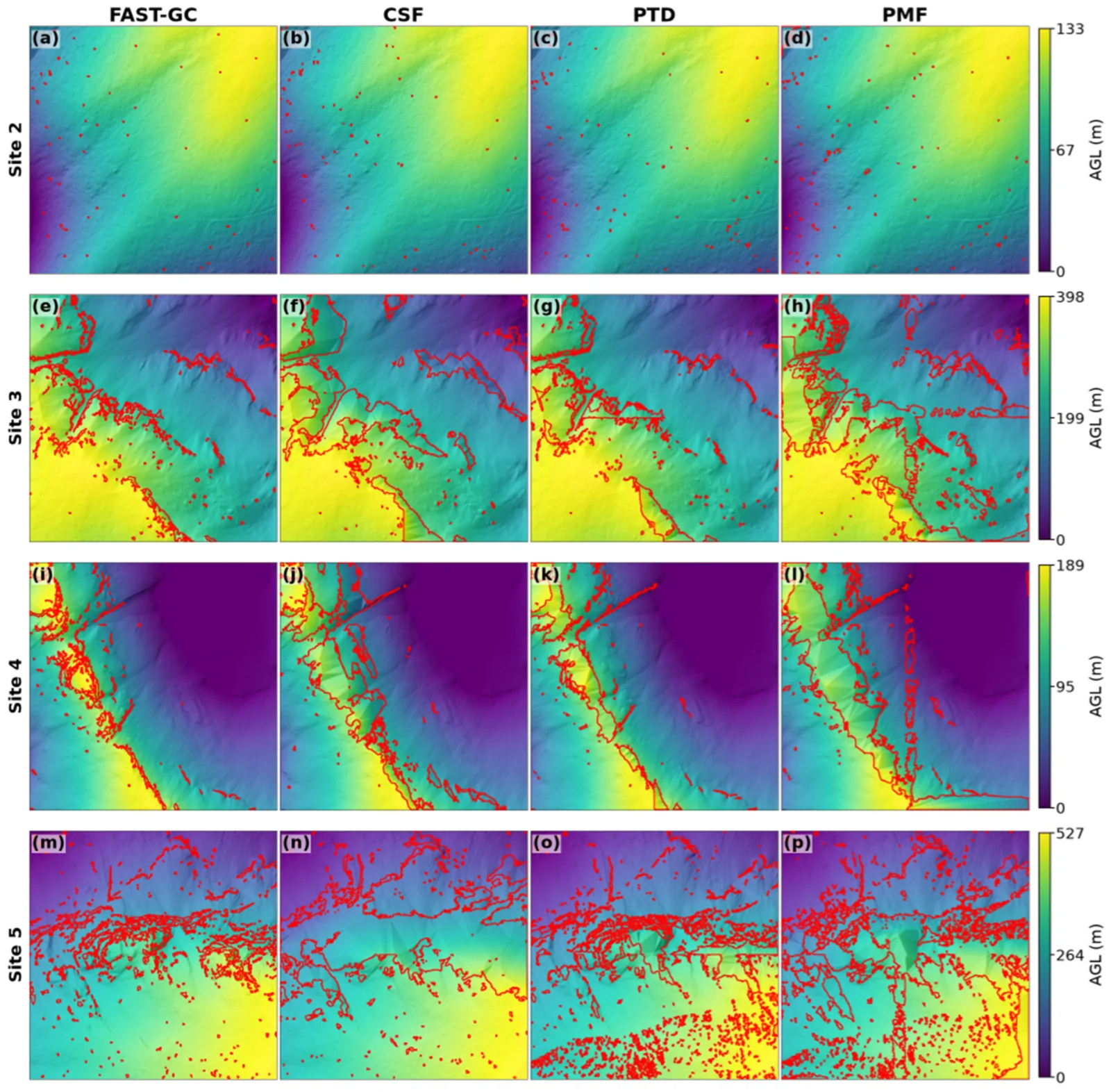
Comparison of 0.5 m DEMs derived from ground points classified by FAST-GC, CSF, PTD, and PMF across benchmark Sites 2–5. Panels represent Site 2 (**a**–**d**), Site 3 (**e**–**h**), Site 4 (**i**–**l**), and Site 5 (**m**–**p**), with the classification methods arranged consistently across columns: FAST-GC (**a**,**e**,**i**,**m**), CSF (**b**,**f**,**j**,**n**), PTD (**c**,**g**,**k**,**o**), and PMF (**d**,**h**,**l**,**p**). DEM elevations are expressed as site-relative above-ground-level (AGL) values, with the minimum terrain elevation normalized to 0 m and a common elevation range applied across methods within each site. Red polygon boundaries delin-eate spatially contiguous regions where the absolute DEM difference from the reference terrain de-rived from benchmark ground points (class = 2) exceeds 0.25 m (|DoD| ≥ 0.25 m). This arrangement enables direct comparison of terrain-surface reconstruction and the spatial distribution of elevation discrepancies among algorithms. Site 1, similar to Site 2, is comparatively flat, with no significant errors observed across algorithms, and is therefore omitted.

Qualitative assessment of high-resolution (HR) 0.5 m DEM (Figure 5) showed that all four methods reproduced the broad terrain morphology of Sites 2–5, yet substantial differences were evident in the spatial distribution and continuity of HR-DEM (0.5 m) residuals relative to the DEM generated from benchmark ground points (see red polygons in Figure 5). On Site 2, DEM reconstruction was highly consistent among all algorithms, with only small and spatially isolated regions exceeding the ±0.25 m DoD threshold set as an evaluation criterion. This agrees with the relatively favorable classification performance observed for Site 2 and indicates that differences in classified ground points had limited influence on the reconstructed terrain surface, given that the topographic complexity is favorable for all GPC algorithms with gentle slope transitions.

However, DoD became substantially more pronounced for GPC algorithms on Sites 3–5. FAST-GC generally preserved the major landforms while limiting DoD to comparatively localized narrow patches (red polygons in Figure 5). In contrast, CSF, PTD, and particularly PMF produced larger and more spatially connected residual regions in the more topographically complex areas. The DoD is especially apparent along steep terrain transitions, ridges, drainage features, and sharp changes in local relief, where the red residual boundaries become increasingly extensive. On Sites 3 and 4, the reference-scale terrain structure remains visually continuous in the FAST-GC DEM, whereas CSF, PMF, and PTD DoDs contain broader zones exceeding the 0.25 m threshold. Site 5 produced the greatest disagreement among algorithms, with extensive residual regions occurring across the major terrain break and adjoining steep surfaces. This observation also supports FAST-GC retaining the larger building structures in urban areas (Site 6, Figure 4).

Overall, the spatial DoD patterns indicate that the principal advantage of FAST-GC was not simply preservation of the overall elevation gradient, which was reproduced by GPC algorithms but improved retention of local terrain morphology at high-resolution DEM (0.5 m). The relatively smaller and less continuous DoD regions for FAST-GC suggest greater agreement with the reference DEM in areas where terrain geometry becomes more complex. Conversely, the increasingly extensive residual boundaries for CSF, PMF, and PTD methods indicate that ground classification differences can propagate into spatially coherent terrain-surface errors, particularly under steep or rapidly varying topographic conditions (see red polygon in Figure 5).

Unlike the counterparts, FAST-GC showed ground OE at extreme topographic transitions and produced more linear polygons (outlined in red) or residuals, thereby producing a DEM that has the highest agreement with the reference DEM originating from benchmark ground points.

### 3.4. Slope-Wise DEM Residual Error Distribution

**Figure 6:**
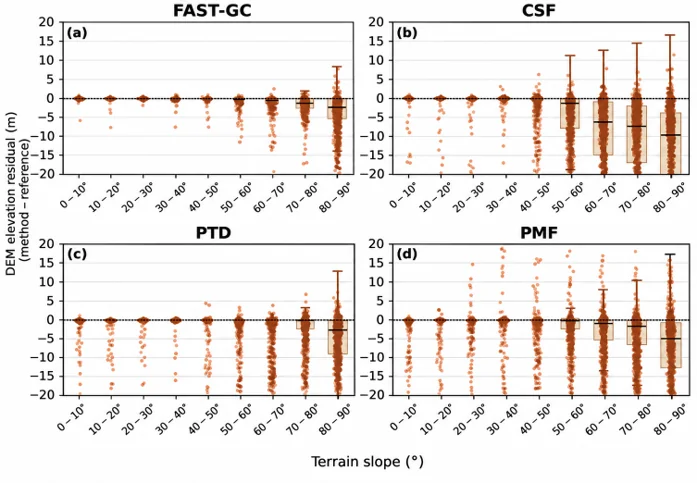
DoD residuals as a function of reference terrain slope for (**a**) FAST-GC, (**b**) CSF, (**c**) PTD, and (**d**) PMF across benchmark Sites 2–5. Residuals were calculated as the method-derived 0.5 m DEM minus the reference DEM derived from the benchmark ground points and grouped into 10° slope intervals from 0–10° to 80–90°. Points show the distribution of cell-level residuals within each slope class, while boxplots summarize their median and interquartile range. The horizontal dotted lines indicate the ±0.25 m DEM-difference threshold. Negative residuals indicate terrain underestimation relative to the reference DEM, whereas positive residuals indicate overestimation.

DoD showed a pronounced dependence on terrain slope, with differences among algorithms increasing progressively toward steeper terrain (Figure 6), results which are consistent with the quantitative evaluation presented in Figure 3 and Figure 4, Table 3. To quantify the DoD residual errors along slopes, we further analyze the DoD distributions for GPC algorithms with slope differences of 10 degrees to 90 degrees. FAST-GC maintained comparatively small residuals through the low-to-moderate slope classes (Figure 6). Its MAE increased from 0.003 m at 0–10° to 0.043 m at 40–50°, while the proportion of cells exceeding the ±0.25 m threshold increased from 0.18% to 1.96%. At 40–50°, the corresponding threshold exceedance rates were substantially greater for CSF (23.31%), PTD (5.68%), and PMF (21.97%).

**Figure 7:**
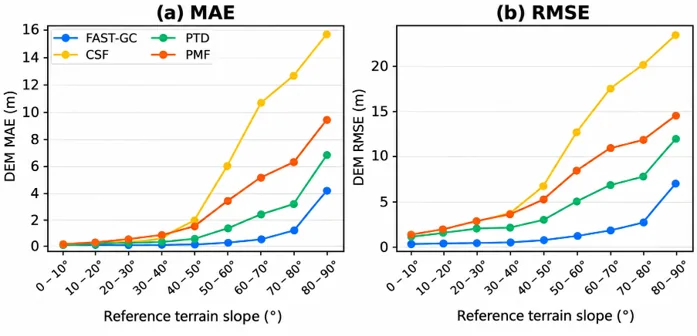
Terrain-surface reconstruction error as a function of reference terrain slope for FAST-GC, CSF, PTD, and PMF across benchmark Sites 2–5. Cell-level DEM residuals from the 0.5 m method-derived and reference DEMs were pooled within 10° reference-slope intervals and summarized using (**a**) mean absolute error (MAE) and (**b**) root mean square error (RMSE). Increasing MAE and RMSE with slope indicates progressive loss of terrain-reconstruction accuracy, while the comparatively lower errors of FAST-GC at steeper slopes demonstrate greater preservation of complex terrain relative to the conventional filtering methods.

The DoD among methods became particularly pronounced above 50°, as reflected by both the residual distributions and the MAE and RMSE trajectories (Figure 7). At 50–60°, FAST-GC achieved an MAE of 0.15 m and RMSE of 0.98 m, compared with 6.06 and 12.76 m for CSF, 1.30 and 5.18 m for PTD, and 3.40 and 8.50 m for PMF, respectively. Only 5.84% of FAST-GC DEM cells exceeded ±0.25 m in this slope class, compared with 64.05% for CSF, 15.36% for PTD, and 45.03% for PMF. At 70–80°, FAST-GC maintained an MAE of 1.00 m, whereas MAE increased to 12.75 m for CSF, 3.12 m for PTD, and 6.23 m for PMF. The corresponding proportions of cells exceeding ±0.25 m were 40.27%, 92.27%, 47.42%, and 79.60%, respectively. At the steepest 80–90° class, FAST-GC retained the lowest MAE (4.13 m) and RMSE (7.28 m), compared with 15.86 and 23.34 m for CSF, 6.90 and 12.09 m for PTD, and 9.59 and 14.66 m for PMF (Figure 7).

Residual direction further revealed a systematic terrain response (Figure 7). Mean residuals became increasingly negative with slope for all methods, indicating progressive underestimation of reference terrain elevation, but this tendency was substantially weaker for FAST-GC. At 60–70°, mean bias was −0.37 m for FAST-GC, compared with −10.75 m for CSF, −2.26 m for PTD, and −4.74 m for PMF. At 80–90°, the corresponding biases were −4.04, −15.75, −6.63, and −8.44 m, respectively.

Together, Figure 6 and Figure 7 quantitatively support the spatial DoD patterns shown in Figure 5: differences among methods were relatively limited on gentle terrain but increased markedly with terrain gradient, with FAST-GC exhibiting the all-time smallest DoD residual distributions and substantially greater preservation of the reference terrain surface under steep conditions.

### 3.5. Extensive Benchmarking of FAST-GC

The controlled five-site benchmark (Table 3; Figure 3, Figure 5, Figure 6 and Figure 7) provided a detailed comparative assessment of FAST-GC against PTD, CSF, and PMF under controlled benchmark sites (n = 5) of unique vegetation and terrain conditions, including steep and highly variable topography. To determine whether the performance observed within controlled benchmark sites—a subset representative of the broader benchmark—held, FAST-GC was subsequently applied to all Jin et al. (2020) [17] benchmark sites (n = 20) at their original point densities to check the algorithm consistency and accuracy (Table 5).

This full-benchmark assessment extended the evaluation across the complete range of available site conditions, with mean canopy cover varying from 0 to 90% and mean terrain slope from 6.0° to 41.5°. FAST-GC achieved macro-average precision, recall, F1-score, and ground-class IoU of 0.99, 0.96, 0.97, and 0.95, respectively. Site-level F1-scores ranged from 0.94 to 0.99, while IoU remained between 0.89 and 0.99, indicating that the GPC accuracy remained consistent beyond the controlled subset (Table 3, Figure 3). CE averaged 0.95%, compared with an OE of 3.89%, indicating that omission of reference ground points was the most prominent classification error. Nevertheless, lower IoUs at original Sites 5 (0.89), 4 (0.90), and 14 (0.91), together with elevated OE at several sites, demonstrate that performance remained sensitive to site-specific conditions. This variability is consistent with the complementary slope-wise DEM analyses (Figure 5, Figure 6 and Figure 7), which showed increasing terrain-reconstruction errors with slope, particularly above 50°, although FAST-GC generally maintained substantially lower errors than its counterparts—CSF, PMF, and PTD. Thus, the complete 20-site evaluation supports the broader consistency of FAST-GC’s point-wise classification performance. MCC exhibited greater site-to-site variability (0.16–0.98), emphasizing that ground-class IoU should be interpreted alongside class composition and complementary discrimination metrics. MCC was undefined for original Site 20 because the reference data contained only ground points.

### 3.6. Point Density Sensitivity Analysis of FAST-GC

**Figure 8:**
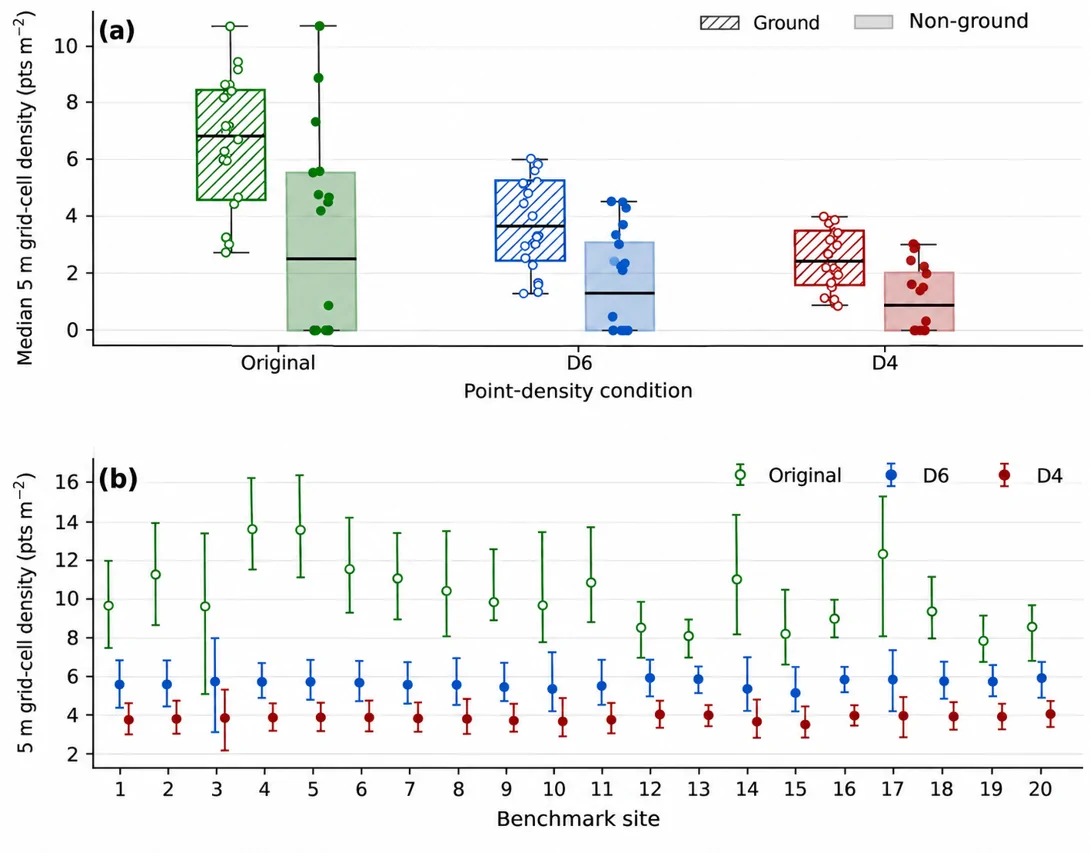
Spatial variation in point density across the Jin benchmark sites (n = 20) under the original, D6, and D4 sampling conditions. (**a**) Distribution of ground and non-ground point densities calculated using a common 5 m × 5 m grid. Boxplots represent the distributions of site-level median grid-cell densities, with individual points representing sites. (**b**) Site-wise spatial variation in total point density, expressed as the median and interquartile range (IQR) of 5 m × 5 m grid-cell densities for each benchmark site.

Given the limited availability of ALS benchmarks acquired across diverse forests and conditions with varying point densities (pts./m^2^), a controlled density sensitivity experiment was conducted using sites (n = 20) from Jin et al. (2020) [17]. For each site, the original point cloud was deterministically and randomly thinned to target densities of approximately 6 pts./m^2^ (D6) and 4 pts./m^2^ (D4). A nested sampling design was applied such that D4 was a subset of D6, and D6 was a subset of the original point clouds. Thinning was not class-aware; therefore, points were retained independently of their ground or non-ground labels, avoiding preferential sampling of either class (Figure 8a). In addition to whole-site pts./m^2^ assessment (Figure 8a), within-site spatial variation in sampling density was characterized using a common 5 m × 5 m grid aligned across the original, D6, and D4 datasets for each site. Total, ground, and non-ground point densities were calculated independently within each grid cell. Cell-level distributions were summarized at the site level using the median and interquartile range (IQR), allowing changes in both overall sampling density and its spatial heterogeneity to be evaluated without disproportionately weighting sites containing different numbers of grid cells.

The density-reduction experiment showed that deterministic thinning effectively decreased ALS sampling density while largely preserving the spatial distribution and ground–non-ground composition of the original point clouds (Figure 8a,b). Across benchmark sites, the median local 5 × 5 m grid-cell density decreased from 9.84 pts./m^2^ for the original datasets to 5.76 pts./m^2^ for D6 and 3.84 pts./m^2^ for D4. Despite this reduction, the spatial variability in point density remained similar among the three conditions, with mean site-level coefficients of variation of 33.90%, 34.17%, and 34.63% for original, D6, and D4, respectively. Ground and non-ground point densities decreased proportionally with thinning (Figure 8a), while the site-wise density distributions retained similar spatial patterns across the three density levels (Figure 8b).

FAST-GC point-wise classification performance remained stable at three different point densities for all 20 benchmark sites (Table 6). The original datasets achieved a precision of 0.99, recall of 0.96, F1-score of 0.97, IoU of 0.95, MCC of 0.85, and balanced accuracy (BA) of 0.97. Comparable performance was maintained after thinning to both D6 and D4, with F1, IoU, MCC, and BA remaining approximately 0.97, 0.95, 0.85, and 0.97, respectively. CE increased modestly from 0.95% for the original datasets to 1.15% at D6 and 1.24% at D4, whereas OE decreased from 3.89% to 3.28% and 3.24%, respectively. These results indicate that FAST-GC ground/non-ground classification was comparatively insensitive to different point densities, producing similar GPC accuracies compared with original point densities, as shown in Table 6.

DEM accuracy also remained comparatively stable under point-density reduction, although a modest increase in terrain error was observed at lower densities (Table 7). MAE increased from 0.07 m for the original datasets to 0.09 m at D6 and 0.11 m at D4, corresponding to an absolute increase of only 0.04 m between the original and lowest-density condition. RMSE similarly increased from 0.38 m to 0.39 m and 0.42 m, while mean bias remained essentially unchanged at approximately −0.04 m across all density levels. A somewhat greater change was observed in the 95th percentile absolute error, which increased from 0.29 m for the original datasets to 0.34 m at D6 and 0.40 m at D4, indicating a modest increase in larger localized elevation errors as sampling density decreased. Overall, these differences suggest that reducing point density to approximately 4 pts./m^2^ had limited influence on aggregate DEM accuracy, with the primary effect being a modest increase in localized rather than systematic terrain error.

**Figure 9:**
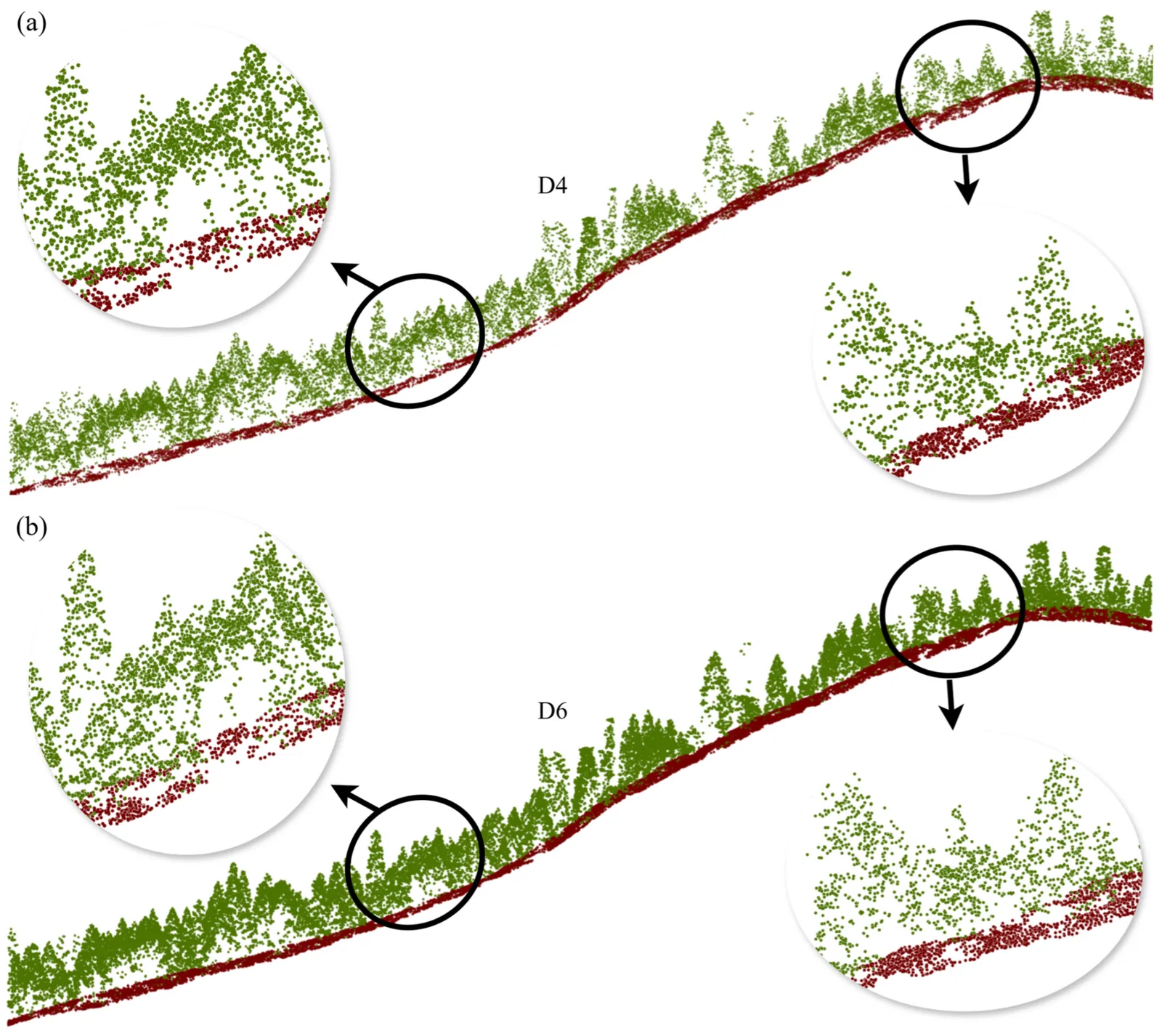
Qualitative evaluation of FAST-GC under reduced ALS point densities. FAST-GC classification results are shown for the same representative terrain profile after deterministic thinning to (**a**) D4 (≈4 pts.m^−2^) and (**b**) D6 (≈6 pts.m^−2^). Red points represent points classified as ground and green points represent non-ground returns (vegetation). Black circles identify representative locations that are enlarged in the corresponding insets to illustrate ground–non-ground separation beneath vegetation and across varying terrain.

The stability of FAST-GC under reduced point density was also evident from visual assessment of the classified point clouds (Figure 9). At both D6 and D4, FAST-GC maintained a spatially coherent and continuous ground surface across densely vegetated and topographically variable terrain, while overlying vegetation was predominantly retained as non-ground. The enlarged views highlight representative locations where ground-return support was locally sparse beneath dense canopy and became progressively weaker following point-density reduction (Figure 9a,b). Despite the reduced number and increased spacing of ground returns, the classified ground points remained coherent with the underlying topographic profile, maintaining ground-surface fidelity across both relatively flat and sloping terrain. This behavior was particularly evident in the canopy-obscured sections highlighted by the insets, where FAST-GC preserved ground–non-ground separation without apparent upward migration of the ground classification into the overlying vegetation or fragmentation of the ground surface. Although D4 contained substantially fewer returns than D6, no pronounced deterioration or systematic displacement of the classified ground surface was visually apparent. These observations complement the quantitative evaluations presented in Table 5 and Table 6 and indicate that FAST-GC maintained stable ground classification at densities ≈ 4 pts./m^2^, including under locally sparse ground-return conditions beneath dense canopy.

It should be noted that FAST-GC has not been systematically evaluated at LiDAR point densities below 4 pts./m^2^. Previous studies have highlighted the importance of sufficient LiDAR sampling density for reliable terrain reconstruction beneath dense forest canopies [81,82]. For example, Leitold et al. (2015) recommend a minimum of 4 pts./m^2^ for dense forests in complex terrain, and White et al. (2013) suggest densities greater than 4 pts./m^2^ for high-resolution DEM and CHM generation under dense canopy conditions [83,84,85]. Consequently, although FAST-GC demonstrated robust GPC performance within the evaluated density range, its suitability to lower-density (<4 pts./m^2^) point clouds should be ascertained before extensive data processing [81].

### 3.7. Cross-Platform Consistency of Ground-Surface Reconstruction

**Figure 10:**
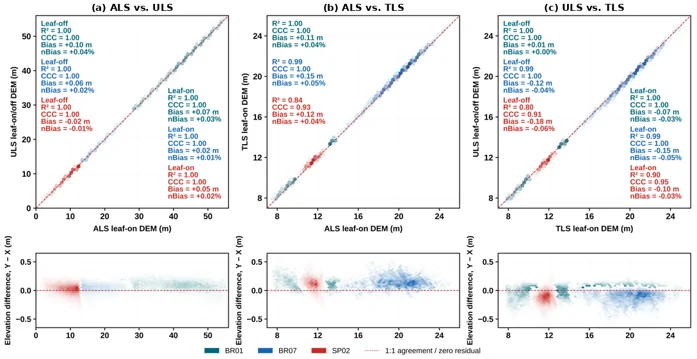
Cross-platform agreement among 0.5 m digital elevation models (DEMs) independently derived from FAST-GC-classified ground points across three temperate mixed-forest plots (BR01, BR07, and SP02, Germany). (**a**) ALS versus ULS, (**b**) ALS versus TLS, and (**c**) ULS versus TLS. Hexagonal bins represent spatially coincident DEM elevations, colored by study plot (BR01, teal; BR07, blue; SP02, red), with leaf-on and leaf-off acquisition comparisons distinguished by the corresponding statistical annotations. Upper panels show elevation agreement relative to the 1:1 reference line, while lower panels show elevation residuals (Y–X) relative to the zero-difference line. Reported statistics include the coefficient of determination (R^2^), concordance correlation coefficient (CCC), absolute bias (m), and normalized bias (nBias, %).

Direct point-wise comparison among independently acquired ALS, ULS, and TLS surveys was not feasible because individual LiDAR returns lacked one-to-one correspondence across acquisitions. The surveys also differed in sampling geometry, point density, acquisition date, and leaf-on/leaf-off conditions (Table 2). Accordingly, cross-platform consistency was evaluated using independently generated 0.5 m DEMs derived from FAST-GC-classified ground points [29]. The assessment was extended to three forest plots (BR01, BR07, and SP02), encompassing 12 LiDAR acquisitions, with comparisons restricted to spatially coincident DEM cells. Across the three plots, the ALS–ULS, ALS–TLS, and ULS–TLS comparisons exhibited strong elevation agreement, with most coefficients of determination (R^2^) and concordance correlation coefficients (CCCs) approaching 1.00 (Figure 10). Elevation biases were generally small, although agreement varied among platforms and plots. The largest departures were observed in SP02 for comparisons involving TLS, particularly under leaf-off conditions, where ALS–TLS and ULS–TLS comparisons yielded lower agreement (R^2^ = 0.84 and 0.80, respectively; CCC = 0.93 and 0.91). Residual distributions remained concentrated near zero across the comparisons, with localized deviations indicating plot- and platform-dependent differences in the reconstructed terrain surfaces (Figure 10).

**Figure 11:**
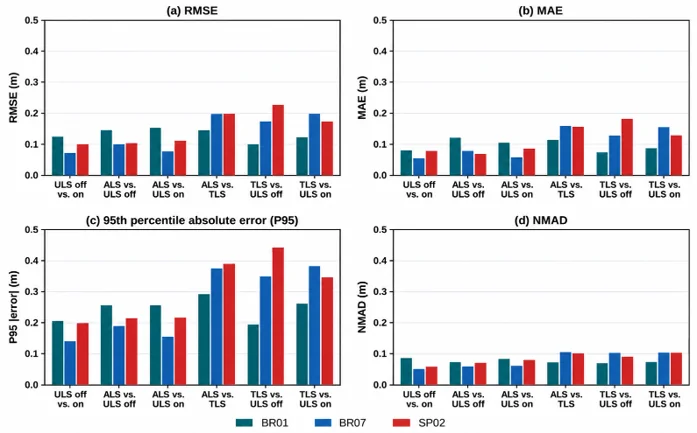
Cross-platform differences among 0.5 m digital elevation models (DEMs) independently derived from FAST-GC-classified ground points across three temperate mixed-forest plots (BR01, BR07, and SP02, Germany). (**a**) Root mean square error (RMSE), (**b**) mean absolute error (MAE), (**c**) 95th percentile absolute elevation error (P95), and (**d**) normalized median absolute deviation (NMAD). Each panel presents six pairwise acquisition comparisons: ULS leaf-off versus leaf-on, ALS versus ULS leaf-off, ALS versus ULS leaf-on, ALS versus TLS, TLS versus ULS leaf-off, and TLS versus ULS leaf-on. Bars distinguish the three study plots (BR01, teal; BR07, blue; SP02, red). All error metrics are expressed in meters and calculated from spatially coincident DEM cells within the corresponding survey overlap areas.

The magnitude and dispersion of DEM elevation differences were further quantified using RMSE, MAE, the 95th percentile absolute error (P95), and normalized median absolute deviation (NMAD) across six pairwise acquisition combinations (Figure 11). Across the three plots, RMSE ranged approximately from 0.07 to 0.23 m, while MAE ranged from 0.06 to 0.18 m. Comparisons between ULS leaf-off and leaf-on acquisitions generally produced the smallest errors (RMSE ≤ 0.12 m), whereas comparisons involving TLS showed greater variability, particularly at BR07 and SP02. The largest RMSE (approximately 0.23 m) and MAE (approximately 0.18 m) occurred for the TLS–ULS leaf-off comparison at SP02, which also exhibited the highest P95 (approximately 0.44 m). In contrast, NMAD remained comparatively low across all acquisition pairs (approximately 0.05–0.11 m), indicating that the larger RMSE and P95 values were associated with more pronounced localized discrepancies rather than uniformly increased residual dispersion. Despite substantial differences in acquisition characteristics and point densities (Table 2), FAST-GC produced broadly consistent terrain elevations across the three forest plots, with the greatest differences occurring in selected TLS-related comparisons (Figure 11).

**Figure 12:**
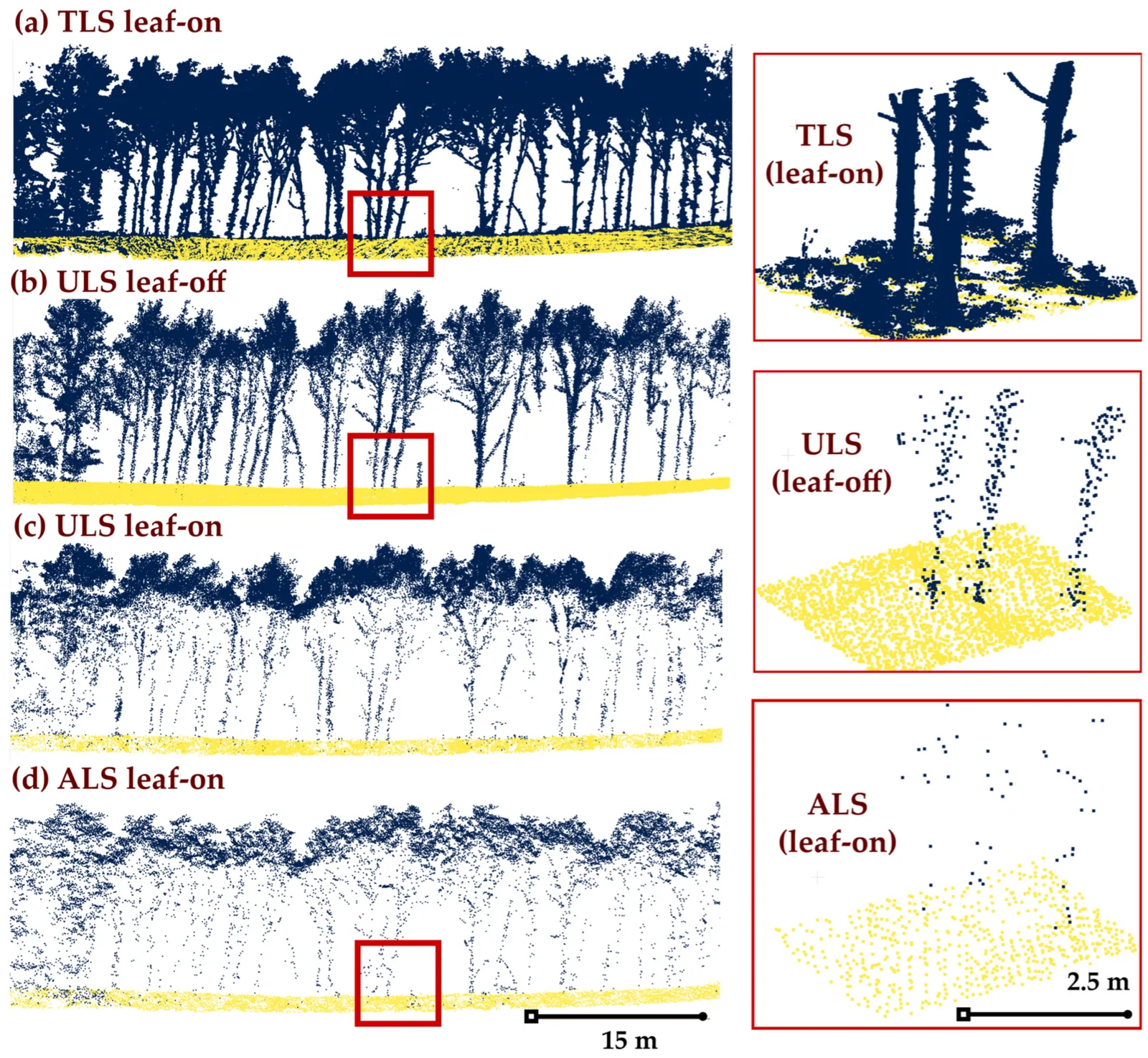
Qualitative comparison of FAST-GC ground classification across concurrent ALS, ULS, and TLS acquisitions at the BR01 temperate mixed-forest plot, Germany. Left panels show vertical point-cloud transects (60 × 5) m for (**a**) TLS leaf-on, (**b**) ULS leaf-off, (**c**) ULS leaf-on, and (**d**) ALS leaf-on, with classified ground points shown in yellow and non-ground points in dark blue. Red rectangles identify representative regions enlarged in the corresponding right-hand panels. The magnified views illustrate differences in ground-point coverage, vegetation penetration, and spatial sampling patterns among acquisition platforms. These differences in terrain-point coverage may influence the interpolation and local agreement of independently generated high-resolution DEM analyses (Figure 10 and Figure 11).

The qualitative assessment at BR01 revealed distinct patterns of ground-point coverage among ALS, ULS, and TLS acquisitions (Figure 12). Despite its substantially higher overall point density (Table 2), TLS exhibited larger gaps in ground observations because its ground-based scanning geometry was susceptible to line-of-sight occlusion by tree stems, shrubs, and understory vegetation, occasionally obscuring entire ground patches (Figure 12a). Nevertheless, FAST-GC retained the available TLS ground observations without apparent contamination from adjacent above-ground objects. In contrast, ALS and ULS exhibited variable ground-return densities beneath forest canopies but maintained comparatively continuous terrain observations under both leaf-on and leaf-off conditions, with greater ground visibility during leaf-off acquisition (Figure 12b–d). These differences were also reflected in the DEM residual distributions (Figure 10): ALS–ULS comparisons showed relatively narrow, continuous residual patterns concentrated near zero, whereas ALS–TLS and ULS–TLS comparisons exhibited greater residual scatter and localized departures from zero, particularly at BR07 and SP02. The corresponding increases in RMSE, MAE, and P95 (Figure 11) are consistent with the effects of incomplete TLS ground coverage, where interpolation at 0.5 m resolution may estimate terrain elevations across occluded regions without direct ground observations (Figure 12a). The strong ALS–ULS agreement further suggests that spatially aligned airborne ground observations could provide complementary terrain constraints for TLS acquisitions, potentially reducing occlusion-related gaps and improving ground points representation in TLS point clouds.

### 3.8. Stage-Wise Contributions and Ablation Analysis of FAST-GC

**Figure 13:**
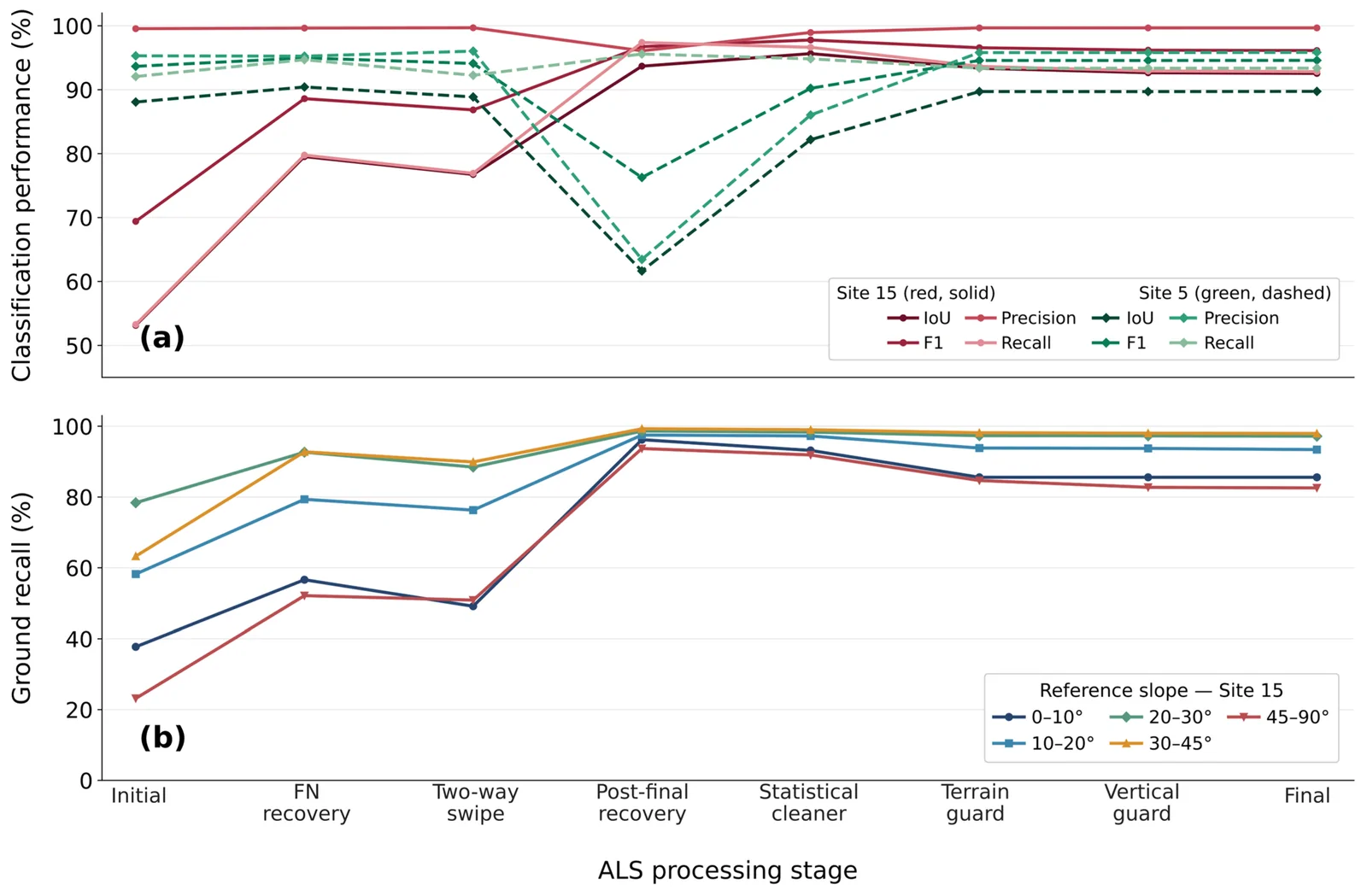
Stage-wise contribution assessment of FAST-GC adaptive ground classification components across sequential airborne laser scanning (ALS) processing checkpoints. (**a**) Evolution of precision, recall, F1-score, and intersection over union (IoU) for Site 15 (solid red lines) and Site 5 (dashed green lines). (**b**) Ground-class recall at Site 15 stratified by reference terrain-slope classes (0–10°, 10–20°, 20–30°, 30–45°, and 45–90°). The analysis identifies performance gains and losses associated with successive ground-recovery, statistical-cleaning, and consistency-checking operations, revealing differences in stage sensitivity and terrain-dependent behavior. The curves represent cumulative stage-wise effects; individual component contributions are evaluated separately through controlled stage-removal ablations. The mathematical background of all FAST-GC stages is documented in Section 2.3.

**Figure 14:**
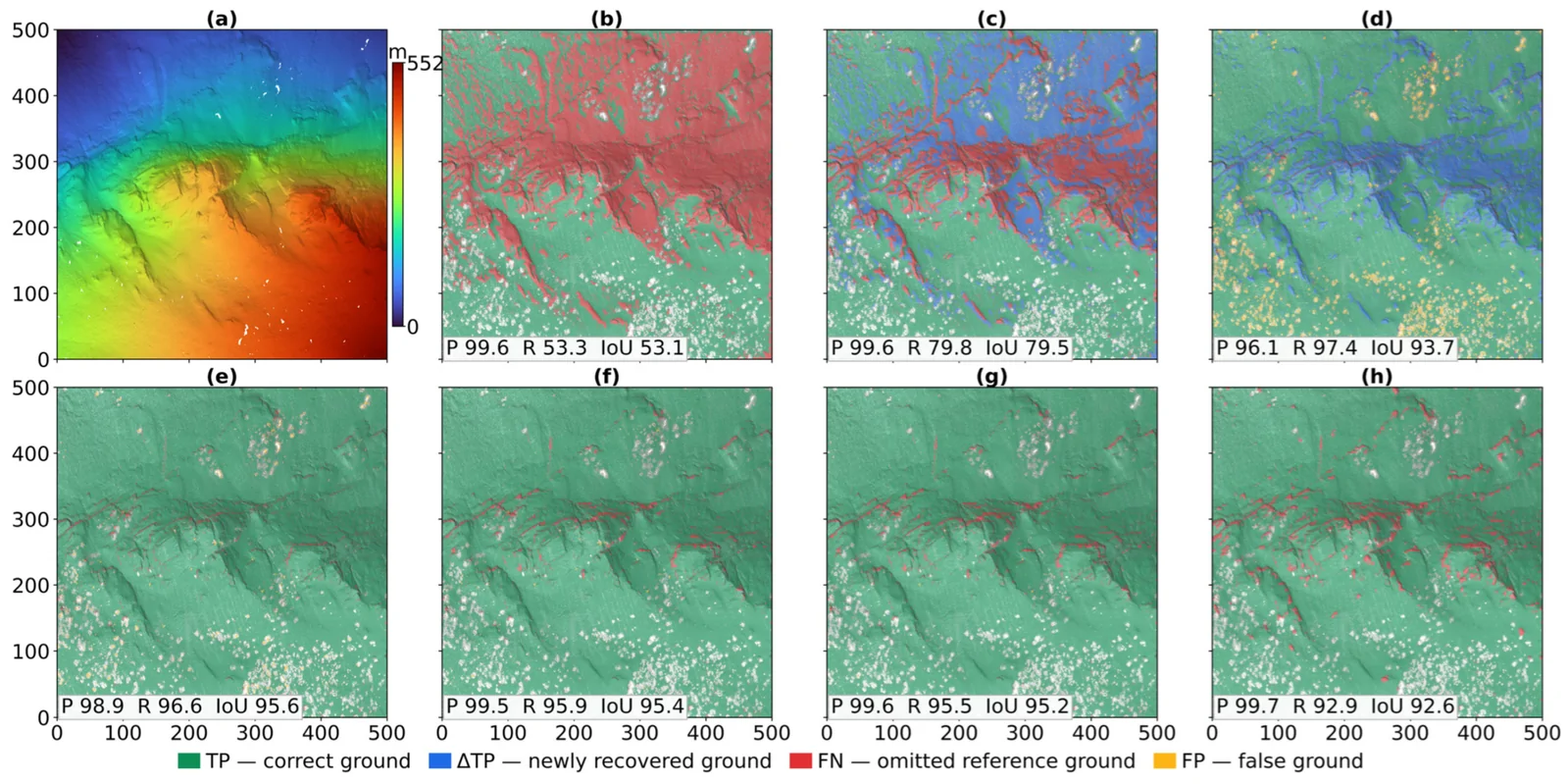
Sequential evolution of FAST-GC ground classification at Site 15 (complex topography): (**a**) reference-ground DEM; (**b**) initial surface; (**c**) false-negative recovery; (**d**) post-final recovery; (**e**) statistical cleaning; (**f**) terrain/blob guard; (**g**) vertical-support guard; and (**h**) final reconciliation. Classification outcomes are overlaid on reference-ground hillshade: green, true positives (TP); blue, newly recovered ground (ΔTP); red, false negatives (FN); and yellow, false positives (FP). Precision (P), recall (R), and IoU are expressed as percentages.

To investigate the contributions of interconnected processing stages within FAST-GC, a hierarchical stage-wise assessment was conducted using two representative benchmark sites: Site 5, characterized by complex vegetation, and Site 15, characterized by complex topography. The analysis examined how successive ground-recovery and refinement operations influence classification performance under contrasting environmental conditions. The same assessment framework was applied to ALS and ULS datasets, with the ALS results presented in Figure 13 and Figure 14 and the corresponding ULS results provided in Supplementary Figure S1.

FAST-GC follows a sequential hierarchical architecture in which each processing stage receives and modifies the classification state produced by preceding stages. At each checkpoint, the cumulative ground classification was evaluated against benchmark point clouds using precision, recall, F1-score, and IoU. For Site 15, ground recall was additionally stratified by reference terrain slope to examine the sensitivity of successive processing stages to topographic complexity. This approach captures the cumulative evolution of classification performance, including intermediate improvements, declines, and subsequent stabilization. Unlike controlled leave-one-stage-out ablation, the assessment preserves all preceding operations and quantifies the immediate changes associated with each successive stage rather than the effect of removing that stage from the complete pipeline.

The ALS results revealed distinct stage-dependent responses between the two benchmark sites (Figure 13a). Site 5 generally maintained high classification performance, although a pronounced intermediate decline occurred during post-final recovery, followed by substantial improvement through subsequent refinement. In contrast, Site 15 exhibited considerably lower initial ground recall and IoU, indicating greater difficulty in identifying ground observations across complex terrain. False-negative recovery improved ground detection, while post-final recovery produced the most pronounced increase in recall, F1-score, and IoU. These contrasting trajectories demonstrate that the contribution of individual processing operations depends on the terrain and vegetation characteristics of the input point cloud.

The slope-stratified assessment of Site 15 (Figure 13b) further demonstrated the influence of topographic complexity on ground classification. Initially, ground recall varied substantially among slope classes, with the steepest terrain exhibiting the greatest omission of reference-ground points. False-negative recovery improved recall across multiple slope categories, while post-final recovery produced a marked increase across all slope classes, substantially narrowing the differences between gentle and steep terrain. Subsequent statistical cleaning and terrain- and vertical-support guarding introduced comparatively smaller changes, indicating that the cumulative classification had approached a more stable state following the principal recovery operations.

The spatial assessment of Site 15 (Figure 14) complements these quantitative findings by illustrating the spatial distribution of classification outcomes throughout the hierarchical sequence. The initial surface exhibited extensive FNs, particularly along steep slopes, ridges, and complex terrain transitions (Figure 14b). FN recovery restored substantial portions of the omitted reference-ground points (Figure 14c), while post-final recovery further improved ground continuity and reduced the spatial extent of OE (Figure 14d). Subsequent statistical cleaning, terrain/blob guarding, and vertical-support guarding produced more localized adjustments to the classification (Figure 14e–g). Final reconciliation retained a spatially continuous representation of the principal terrain features, thereby reducing the artefacts at tile boundaries, although residual OE remained concentrated along selected complex terrain transitions (Figure 14h). These spatial patterns demonstrate that FAST-GC hierarchical ground recovery occurs during intermediate stages, whereas later operations primarily refine the balance between ground OE and CE.

Importantly, the stage-wise results also reveal that improvements in ground recall do not necessarily persist unchanged through the final processing sequence. At Site 15, recall increased from 53.3% after initial surface construction to 79.8% following false-negative recovery and reached 97.4% after post-final recovery, before declining to 92.9% after final reconciliation (Figure 14). This reduction, accompanied by an increase in precision from 96.1% to 99.7%, indicates a shift toward more conservative ground classification during subsequent refinement. Thus, the final stages should not be interpreted as uniformly improving all performance metrics; rather, they modify the balance between retaining legitimate ground points and suppressing incorrectly classified non-ground observations.

Despite their contrasting intermediate trajectories, both ALS sites exhibited comparatively stable classification performance during the later processing stages (Figure 13). The corresponding ULS assessment (Supplementary Figure S1) extends the hierarchical evaluation to another LiDAR acquisition modality, allowing stage-dependent responses to be examined under different sampling characteristics. Collectively, these analyses demonstrate how FAST-GC progressively constructs and refines its ground classification through interconnected operations, while highlighting the dependence of individual stage contributions on environmental complexity. It should be noted that TLS mode shares the same mathematical background as ALS and ULS, with some additional sensor specific filter that deals with extreme occlusion cases using the radial spread function. Furthermore, reference TLS ground points were not available to perform the extensive stage-wise assessment.

The hierarchical stage-wise assessment provides an interpretable evaluation of the internal classification process but should be distinguished from controlled algorithmic ablation. Because each downstream stage operates on the classification state inherited from earlier operations, omitting an intermediate stage may alter the behavior of all subsequent stages. Consequently, differences between successive checkpoints characterize cumulative classification changes but do not independently establish the effect of removing a module from the complete algorithm.

## 4. Discussion

### 4.1. Terrain Complexity and Algorithmic Behavior

The five benchmark ALS sites’ evaluation demonstrated that differences among GPC algorithms were relatively limited under moderate terrain conditions but increased substantially with topographic complexity (Table 3; Figure 3, Figure 4, Figure 5, Figure 6 and Figure 7) [64,86]. This departure was driven primarily by increasing omission of legitimate ground points by the GPC algorithms and was subsequently expressed as higher terrain-surface residuals with increasing slope [86]. The effect was particularly pronounced above 50°, with FAST-GC maintaining substantially smaller DEM residuals than CSF, PTD, and PMF (Figure 6 and Figure 7). These findings indicate that terrain complexity, rather than overall point-wise classification alone, provides an important test of GPC stability over diverse topographic regimes.

GPC algorithms are generally organized around a combination of filtering mathematical frameworks, and their response to increasing terrain complexity is consequently influenced by the parameters governing those mathematical frameworks [86]. PMF progressively enlarges a morphological window and applies elevation-difference thresholds related to terrain slope; therefore, the balance between preserving rapid terrain variation and removing above-ground objects depends on the selected spatial scale and elevation thresholds [30,73,87]. PTD instead constructs an initial terrain surface from seed points and progressively densifies the TIN (progressive TIN densification) in concordance with user-defined parameters, making its performance dependent on the initial terrain seeds and the spatial, distance, and angular thresholds used during densification [18,72]. Similarly, CSF approaches the problem through an inverted point cloud and a simulated cloth, for which cloth resolution, rigidity, classification threshold, and associated terrain-fitting controls influence the ability of the simulated surface to conform to local topography, and extensive optimization becomes an essential step to obtain the desired accuracy over a given topography regime [29,62]. Previous studies have reported corrosion of legitimate ground points in steep, discontinuous, densely vegetated, or otherwise-heterogeneous terrain, consistent with the increasing ground OE and terrain-surface errors observed for the reference algorithms in the present evaluation [40,42,78,86]. Although both CSF and FAST-GC employ vertical inversion, inversion serves fundamentally different purposes in the two frameworks. CSF fits a simulated deformable cloth to the inverted point cloud, with the simulation governed by grid resolution (GR), time step (dT), rigidness (RI), and the steep-slope fit factor (ST), together with distance-based thresholds for final point classification and steep-slope post-processing [62]. FAST-GC instead uses inversion to expose terrain-facing LiDAR returns and progressively consolidates these initial ground candidates through statistical voting and neighborhood consensus [87]. The resulting terrain-support manifold is, therefore, obtained directly from original LiDAR point clouds rather than through simulation of an intermediate cloth surface, avoiding the cloth-specific parameterization required by CSF.

FAST-GC does not assume that the initially reconstructed terrain surface is complete. The initial stage prioritizes terrain support while rejecting clearly above-ground points, followed by complementary recovery and quality-control operations. Missing ground points are recovered where neighboring observations support terrain continuity, whereas isolated or potentially FP ground points are evaluated through terrain-consistency constraints. This sequential refinement addresses weakly sampled terrain, depressions, abrupt topographic transitions, detached ground points, and locally contaminated terrain components according to their spatial and geometric context, without requiring scene-specific optimization of multiple user-defined parameters. Such refinement is particularly relevant in complex topographic environments, where previous studies have documented increased ground classification errors along steep slopes, sharp ridges, and abrupt terrain changes [36,86]. The resulting behavior provides a systematic explanation for the maximum ground-point retention and terrain-surface reconstruction observed for FAST-GC with increasing topographic complexity and sensor-platform variability, as investigated in this study (Table 3, Table 4 and Table 5; Figure 3, Figure 4, Figure 5, Figure 6, Figure 7, Figure 8 and Figure 9).

### 4.2. Adaptive Spatial Support and Sampling-Density Assessment

Point density (pts./m^2^) directly influences the topographic detail available in LiDAR data [88]. Higher pts./m^2^ can preserve finer-scale terrain morphology and micro-topography, thereby increasing the topographic complexity that GPC algorithms may struggle to resolve under a given set of fixed parameters [88]. As pts./m^2^ decreases, some fine-scale topographic details may be lost, while broader terrain morphology remains observable, and this can be better for some GPC algorithms to operate on a more simplified version of point clouds, e.g., ALS [88]. Therefore, a robust GPC algorithm should preserve the topographic detail available in the LiDAR data across varying pts./m^2^ while maintaining reliable ground points [73,85]. The spatial scale used for terrain assessment is also important because it determines how much local topographic variation is represented during GPC. Previous work showed that smaller neighborhoods can better preserve fine local topography when sufficient ground points are available, whereas larger neighborhoods can smooth the resulting DTM [87]. Reductions in pts./m^2^ can, therefore, reduce the ground-point budget, thereby increasing the distance among available ground points. Previous studies have shown that ground-filtering performance can vary with point densities, and parameter optimization becomes an essential part of GPC [73]. For example, adaptive PTD approaches have incorporated point density and terrain slope into grid construction and densification thresholds to improve terrain representation under varying sampling conditions. Recent benchmarking has likewise demonstrated interactions among sampling density, algorithm configuration, and ground-filtering performance [73].

FAST-GC addresses variation in pts./m^2^ by adapting the local neighborhood used for terrain inference to the observations themselves—a data-driven approach (Equations (1)–(2)). Dataset-level sampling statistics establish the initial neighborhood scale, while tile-level statistics provide bounded local adjustments relative to the dataset context. The local neighborhood can, therefore, expand where terrain observations become sparse and contract where greater point support is available, while remaining within sensor-specific bounds. This adaptation enables FAST-GC to maintain sufficient local terrain context across varying pts./m^2^ without requiring scene-specific parameter calibration.

The systematic density-reduction experiment provides empirical support for the stability of this integrated formulation (Table 6 and Table 7; Figure 8 and Figure 9). Reducing the median ALS point density from approximately 9.84 pts./m^2^ in the original datasets to 3.84 pts./m^2^ at D4 produced only small changes in point-wise classification metrics, while DTM MAE increased from 0.07 to 0.11 m (Table 7). Nevertheless, the topographic inset-map examples in Figure 8 show that terrain continuity remained unchanged beneath vegetation and slope areas, even as the overall pts./m^2^ decreased from the D6 to the D4 subset (Figure 8a,b). These findings are consistent with previous studies, showing that GPC performance can vary across pts./m^2^ and can also depend on user-defined parameters and topographic conditions [50,73]. Moreover, this investigation indicates that for LiDAR surveys acquired at substantially higher pts./m^2^, the data can be reduced to a lower pts./m^2^ (D4 or D6) for DTM-oriented processing while maintaining reliable ground-point classification and terrain reconstruction, thereby providing a practical approach for reducing the computational burden of FAST-GC [87].

### 4.3. Sensor-Aware Terrain Reconstruction Across ALS, ULS, and TLS

Sampling density alone does not fully characterize the information available for terrain elevation, because LiDAR platforms differ substantially in acquisition geometry and the resulting spatial distribution of returns [29,88,89]. ALS generally provides predominantly downward-looking observations over comparatively broad footprints, whereas ULS can provide denser and more locally variable sampling because of lower flight altitude and acquisition configuration [88]. TLS represents a more distinct geometry, with predominantly lateral observations, strong range-dependent sampling, occlusion, and substantial spatial anisotropy within forest environments [89,90]. Consequently, similar nominal point densities across platforms do not necessarily provide equivalent terrain support, and transferability of a GPC framework should, therefore, be evaluated not only across density levels but also across contrasting acquisition geometries.

The cross-platform assessment complements the systematic ALS density experiment by testing this additional source of variability. FAST-GC produced closely corresponding terrain surfaces from independently acquired ALS, ULS, and TLS observations despite substantial differences in sampling geometry and local point distribution (Figure 10, Figure 11 and Figure 12). The correspondence was particularly important in areas where terrain observations were incompletely sampled by one or more platforms, indicating that reconstruction was not dependent exclusively on the relatively regular airborne sampling conditions represented in the ALS development data. Rather than applying an identical spatial configuration to all sensors, FAST-GC retains a common terrain-first principle while adapting the scale and form of terrain-support estimation to the acquisition mode. ALS and ULS share the airborne terrain-support pathway with independently adapted spatial support, whereas TLS employs a dedicated inverted terrain-voting pathway designed for its strongly anisotropic and occlusion-dominated sampling geometry.

These results provide evidence that the underlying terrain-support concept can transfer across substantially different LiDAR acquisition geometries while allowing the implementation pathway to respond to sensor-specific sampling characteristics. This distinction is important because cross-platform consistency does not require the observations themselves to be geometrically equivalent; instead, it requires independently sampled terrain evidence to converge toward a consistent representation of the underlying surface (Figure 12).

### 4.4. Reference-Label Uncertainty and Implications for GPC Benchmarking

Evaluation against the OpenGF stress test produced a markedly different algorithm ranking from the controlled ALS Jin et al. (2020) [17] benchmark (Table 8), highlighting the influence of reference-label provenance on GPC assessment. FAST-GC retained nearly all reference ground points, achieving the highest mean recall (0.97) and lowest OE (2.65%), but exhibited lower mean precision (0.53) and substantially higher reference-based CE (46.86%). Conversely, PMF showed the strongest reference-based mean F1 (0.75), IoU (0.60), and MCC (0.69). Thus, the principal difference was not failure to recover the reference ground population but the substantially larger ground population identified by FAST-GC beyond that represented by OpenGF.

**Figure 15:**
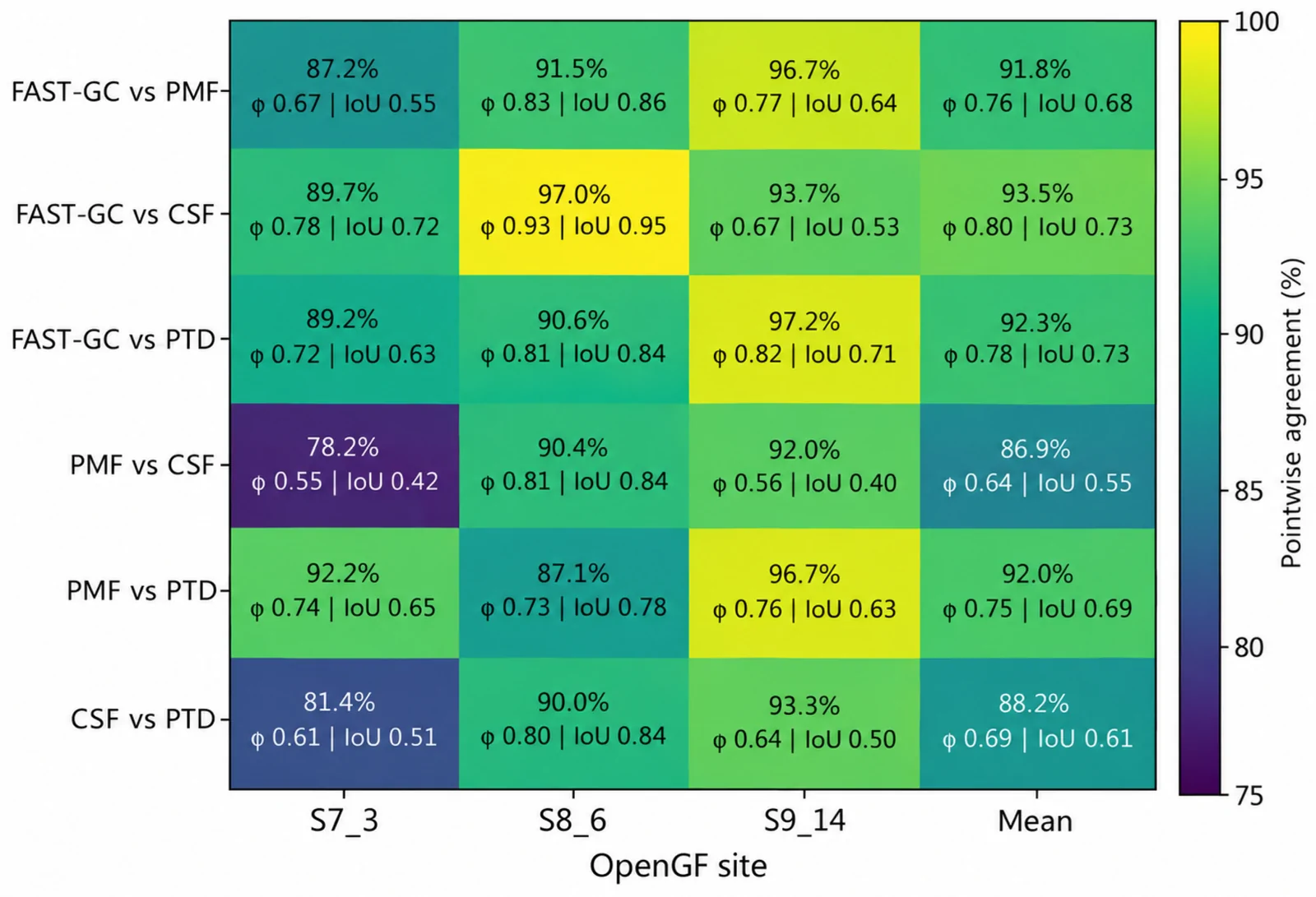
Pairwise agreement among FAST-GC, PMF, CSF, and PTD classifications for the OpenGF mountainous scenes S7_3, S8_6, and S9_14. Cell values report positive agreement (%), with the corresponding Matthews correlation coefficient (MCC) and intersection over union (IoU). The final column reports the mean across the three scenes. The analysis is independent of the supplied OpenGF reference labels and therefore characterizes the degree to which independently applied ground classification algorithms identify a common ground-point population.

Pairwise comparison of the independently generated classifications provides important context for this discrepancy (Figure 15). Despite its high reference-based CE, FAST-GC showed mean positive agreement of 91.8%, 93.5%, and 92.3% with PMF, CSF, and PTD, respectively. These values were comparable to the agreement among the three established algorithms themselves, which ranged from 86.9% for PMF–CSF to 92.0% for PMF–PTD. FAST-GC, therefore, did not identify an entirely distinct ground-point population despite its substantially greater disagreement with OpenGF high-quality ground labels. Importantly, this inter-algorithm agreement does not establish that the additional FAST-GC ground returns were necessarily correct; rather, it demonstrates that disagreement over the extent of the ground population was shared among independently applied GPC algorithms and that reference-based algorithm ranking can be strongly influenced by the composition of the benchmark labels.

OpenGF provides substantial geographic and topographic diversity and is an important resource for development and evaluation of ground-filtering methods [60]. However, its reference labels were constructed primarily from pre-existing classifications in publicly available ALS datasets, followed by class consolidation and repeated quality checking; the published methodology does not describe exhaustive point-wise manual re-annotation or provide an independent quantitative assessment of ground-label completeness. This distinction is particularly relevant in densely vegetated mountainous terrain, where the OpenGF authors reported that ground returns can be extremely sparse [48]. Accordingly, the high reference-based CE of FAST-GC should be interpreted as disagreement with the OpenGF rather than, by itself, as confirmation that all additional ground predictions are FPs. More broadly, these results demonstrate the importance of considering reference provenance alongside conventional point-wise metrics, particularly when evaluating GPC algorithms in sparsely sampled and topographically complex environments. More broadly, geographically extensive benchmarks provide essential environmental diversity but can make exhaustive verification of individual ground returns impractical. GPC evaluation should, therefore, consider reference provenance together with conventional point-wise metrics and terrain-surface behavior, particularly where sparsely represented terrain observations determine apparent CE or OE.

### 4.5. Computational Performance, Operational Applicability, and Limitations

**Figure 16:**
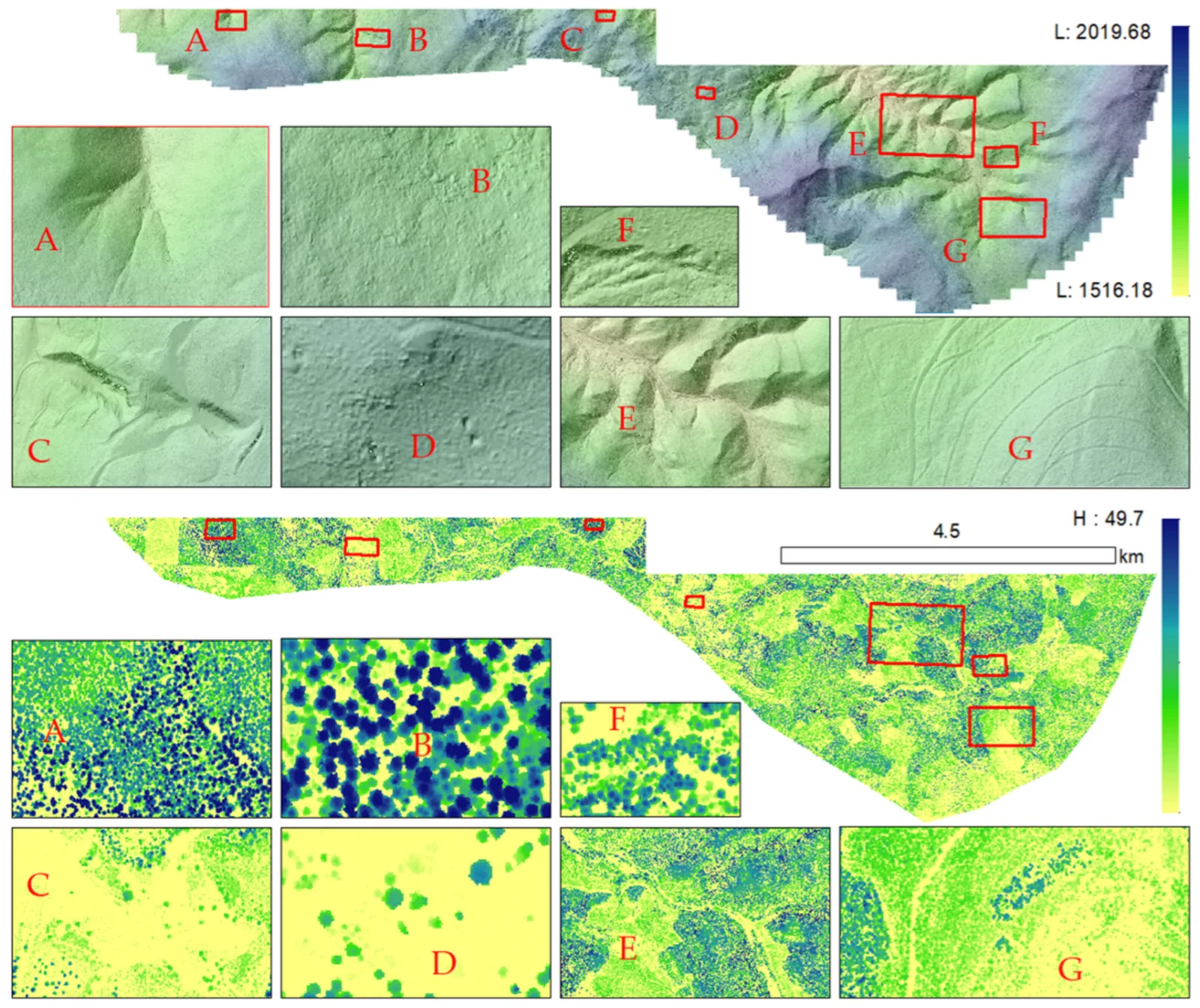
Large-area stability assessment of FAST-GC across approximately 23 km^2^ of heterogeneous forest at Lubrecht Experimental Forest, Montana, USA [67]. The upper panel presents the FAST-GC-derived high-resolution digital elevation model (HR-DEM) at 0.5 m spatial resolution, with representative locations (**A**–**G**) enlarged to illustrate terrain morphology across the processing extent. The subsets include channel-like and convergent terrain (**A**), rough local terrain beneath tall and heterogeneous canopy (**B**), irregular local relief (**C**), a localized depression-like feature (**D**), broader valley-and-ridge morphology with concave and convex hillslope forms (**E**), localized incised terrain (**F**), and narrow linear features consistent with unpaved roads or tracks (**G**). The lower panel presents the corresponding 0.5 m canopy height model (CHM) and the same enlarged locations, illustrating contrasting vegetation and canopy conditions overlying these terrain configurations. The spatially continuous DEM and CHM, together with geographically distributed subsets (**A**–**G**), illustrate large-area processing across heterogeneous canopy and terrain conditions without visually apparent systematic tile-boundary artifacts. The CHM was generated at the same 0.5 m spatial resolution using the pit-free algorithm implemented within the FAST-GC processing workflow (Figure 1).

Beyond classification accuracy, operational applicability of GPC algorithms depends on their ability to process spatially extensive point-cloud datasets reliably under complex input-data conditions. FAST-GC addresses this requirement through buffered tile-wise processing and core-area merging, allowing local terrain evidence to extend across tile boundaries while avoiding duplication in the final output. Successful processing of all 382 evaluated tiles and completion of each downstream processing stage without failure demonstrate the operational applicability of the framework to large-area ALS processing (Figure 16). The modular workflow further separates GPC from downstream terrain-product generation, allowing classified point clouds to be reused for DEM generation, height normalization, and canopy-product derivation without repeating GPC (Figure 1).

Practical portability is additionally supported by implementation of the FAST-GC outside the reference software. Following its public release on 23 April 2026 (https://pypi.org/project/fastgc/, accessed on 15 September 2026), FAST-GC was independently implemented within the open-source projax3d geospatial framework as a GPC algorithm alongside established algorithms such as PMF and CSF (https://projax-6169a6.gitlab.io/api/analysis/, accessed on 15 September 2026). Although independent software implementation does not constitute additional validation of classification accuracy, it indicates that the underlying terrain-voting formulation can be reproduced outside the original Python implementation and incorporated into alternative point-cloud processing environments.

The large-area assessment at Site 9 (Lubrecht Experimental Forest, Montana, USA) provided additional evidence of the operational stability of FAST-GC across heterogeneous forest and terrain conditions (Figure 16). The 0.5 m high-resolution digital elevation model (HR-DEM) retained diverse topographic features beneath contrasting canopy conditions, as illustrated by the corresponding canopy height model (CHM). Representative subsets included convergent slopes and channel-like terrain (Figure 16A), rough terrain beneath tall and heterogeneous canopy (Figure 16B), irregular local relief under relatively sparse canopy (Figure 16C), localized depression-like features (Figure 16D), valley-and-ridge systems with concave and convex hillslopes (Figure 16E), incised terrain (Figure 16F), and narrow linear features potentially associated with ditches, unpaved roads, or tracks (Figure 16G). These observations indicated that the reconstructed terrain retained distinct morphological characteristics across varying forest structures, although the geomorphic origins of individual features were not independently verified.

The HR-DEM and CHM exhibited no visually apparent tile-boundary artifacts or systematic surface discontinuities across the examined subsets. All 382 tiles, covering approximately 23.9 km^2^ of nominal processing area, were successfully processed using the same buffered tile-run-merge workflow without scene-specific parameter calibration. The framework also supported automated generation of user-selected downstream products. Collectively, these findings demonstrated successful large-area execution and visually coherent terrain reconstruction across the evaluated forest and topographic conditions.

Several limitations nevertheless remained. The urban stress test showed that FAST-GC’s preference for retaining coherent terrain support could increase commission error (CE) where anthropogenic surfaces exhibited terrain-like spatial continuity, illustrating the competing objectives of ground retention and above-ground object rejection in structurally complex environments (Table 4; Figure 4). PTD showed comparatively stronger ground/non-ground discrimination in the evaluated urban setting, although performance varied among individual metrics. Furthermore, cross-platform assessment was restricted to the airborne laser scanning (ALS), uncrewed aerial vehicle laser scanning (ULS), and terrestrial laser scanning (TLS) configurations examined in this study and did not constitute exhaustive validation across scanner geometries, vegetation structures, or sampling regimes (Figure 11 and Figure 12).

## 5. Conclusions

This study introduced FAST-GC, an automated and scalable ground-point classification (GPC) algorithm designed for heterogeneous LiDAR datasets without scene-specific parameter calibration. FAST-GC integrated robust terrain-support estimation, adaptive spatial support, terrain-consistency classification, topology-constrained recovery, and post-classification quality control within a unified GPC framework. Rather than relying on a fixed set of user-defined parameters across contrasting terrain and sampling conditions, the algorithm derived and adjusted its spatial support from the characteristics of the observations while maintaining sensor-specific bounds.

Across the controlled ALS comparison, FAST-GC maintained high ground retention while limiting commission error (CE), with differences among the evaluated GPC algorithms becoming increasingly pronounced as topographic complexity increased. This behavior was particularly evident in the slope stress test, where FAST-GC achieved a digital terrain model (DTM) mean absolute error (MAE) of 1.00 m at slopes of 70–80°, compared with 3.12–12.75 m for cloth simulation filtering (CSF), progressive morphological filtering (PMF), and progressive TIN densification (PTD). The extended evaluation across 20 ALS sites further demonstrated stable point-wise classification performance, with mean precision, recall, F1-score, intersection over union (IoU), and Matthews correlation coefficient (MCC) of approximately 0.99, 0.96, 0.97, 0.95, and 0.85, respectively. Together, these results indicated that the principal contribution of FAST-GC under complex terrain was its ability to retain legitimate ground points while preserving the morphology of the reconstructed terrain surface. The stage-wise assessment across representative complex-vegetation and complex-topography sites further documented how successive recovery and refinement operations influenced ground retention and classification performance. FAST-GC also maintained stable performance as point density decreased. Reducing the median density from 9.84 to 3.84 pts./m^2^ produced little change in point-wise classification performance, with F1 ≈ 0.97, IoU ≈ 0.95, and MCC ≈ 0.85, while DTM MAE increased from 0.07 to 0.11 m. These findings demonstrated that FAST-GC maintained reliable ground-point classification and terrain reconstruction across the evaluated range of point densities without density-specific user calibration.

Cross-platform assessment further demonstrated the applicability of the framework beyond ALS. Independently reconstructed 0.5 m terrain surfaces from ALS, ULS, and TLS showed strong correspondence despite substantial differences in acquisition geometry and sampling conditions (R^2^ and concordance correlation coefficient (CCC) ≥ 0.99; RMSE = 0.10–0.16 m). These results supported the transferability of the terrain-reconstruction framework across the evaluated platforms without scene-specific parameter calibration, although the limited number of cross-platform observations did not constitute exhaustive validation of sensor independence. Large-area processing of 382 ALS tiles covering approximately 23.9 km^2^ additionally demonstrated stable tile-wise execution and automated terrain-product generation, supporting the operational applicability of the tile-run-merge architecture for large-area processing.

The OpenGF assessment also highlighted an important consideration for GPC benchmarking. FAST-GC achieved a mean ground recall of 0.97 and an omission error (OE) of 2.65% but lower reference-based precision because it classified substantially more points as ground than were represented by the supplied reference labels. Pairwise agreement with a supplied classification alone could not determine whether these additional points represented classification errors or legitimate terrain observations. Consequently, these findings emphasized the importance of considering reference-label provenance alongside point-wise agreement, spatial terrain continuity, and the accuracy of the resulting terrain surface, particularly in topographically complex and vegetated environments.

## Figures and Tables

**Table 3:** Point-wise ground-point classification performance of FAST-GC and three reference algorithms (CSF, PTD, and PMF) across five controlled ALS benchmark sites. Evaluation metrics include precision, recall, F1-score (F1), intersection over union (IoU), Matthews correlation coefficient (MCC), commission error (CE, %), and omission error (OE, %).

Site	Algorithm	Precision	Recall	F1	IoU	MCC	CE (%)	OE (%)
Site1	**FAST-GC**	**0.99**	**0.97**	0.98	0.96	0.96	0.85	2.87
	PTD	**0.99**	0.82	0.90	0.81	0.85	**0.10**	18.46
	CSF	**0.99**	0.99	**0.99**	**0.97**	**0.98**	1.27	**1.29**
	PMF	**0.99**	0.90	0.94	0.89	0.91	0.52	10.33
Site2	**FAST-GC**	**0.99**	0.99	0.99	**0.99**	0.98	0.47	0.41
	PTD	**0.99**	0.82	0.90	0.82	0.76	**0.15**	17.96
	CSF	**0.99**	**1.00**	**1.00**	**0.99**	**0.99**	0.65	**0.13**
	PMF	**0.99**	0.97	0.98	0.97	0.95	0.30	2.93
Site3	**FAST-GC**	**0.99**	**0.96**	**0.98**	**0.96**	**0.65**	0.06	**3.81**
	PTD	0.99	0.77	0.87	0.77	0.31	**0.01**	23.12
	CSF	0.99	0.79	0.88	0.79	0.32	0.07	20.99
	PMF	**1.00**	0.72	0.83	0.72	0.27	0.04	28.32
Site4	**FAST-GC**	**1.00**	**0.97**	**0.98**	**0.97**	**0.59**	0.04	**2.89**
	PTD	**1.00**	0.75	0.86	0.75	0.22	**0.01**	25.07
	CSF	**1.00**	0.85	0.92	0.85	0.29	0.05	14.73
	PMF	**1.00**	0.75	0.86	0.75	0.22	0.03	25.18
Site5	**FAST-GC**	**0.99**	**0.93**	**0.96**	**0.92**	**0.85**	0.33	**6.82**
	PTD	**0.99**	0.69	0.82	0.69	0.57	**0.04**	31.00
	CSF	**0.99**	0.70	0.82	0.69	0.57	0.50	30.38
	PMF	**0.99**	0.63	0.77	0.63	0.51	0.19	37.35
**Macro-average**	**FAST-GC**	**0.99**	**0.96**	**0.98**	**0.96**	**0.81**	0.35	**3.36**
	PTD	**0.99**	0.77	0.87	0.77	0.54	0.06	23.12
	CSF	**0.99**	0.86	0.92	0.86	0.63	0.51	13.50
	PMF	**0.99**	0.79	0.88	0.79	0.57	0.22	20.82

Notes: Precision, Recall, F1, IoU, and MCC are reported as proportions rounded to two decimal places. Bold values indicate the best result within each benchmark site for the corresponding metric. Best-performing methods were identified from the unrounded results.

**Table 4:** Point-wise classification performance of FAST-GC, PTD, CSF, and PMF on the urban and extreme-topography ALS stress-test sites.

Test	Method	Precision	Recall	F1	IoU	MCC	CE (%)	OE (%)
Urban (Site 6)	FAST-GC	0.86	0.99	0.92	0.86	0.87	13.17	0.27
	PTD	**0.95**	0.84	0.89	0.81	0.83	**4.72**	15.92
	CSF	0.91	1.00	**0.95**	**0.91**	**0.92**	9.21	0.12
	PMF	0.78	**1.00**	0.87	0.78	0.78	22.45	**<0.01**

**Table 5:** Point-wise FAST-GC classification performance across the Jin et al. (2020) [17] benchmark complete sites (n = 20). Ground was treated as the positive class. CE, commission error; OE, omission error. N/A indicates that MCC is undefined for Site 20 because this bare-earth site contains no reference non-ground points. Macro-averages use all 20 sites for defined metrics; MCC is averaged across the 19 sites for which it is defined. Displayed values are truncated to two decimal places (not rounded).

Site	Precision	Recall	F1	IoU	MCC	CE (%)	OE (%)
Site 1	0.99	0.99	0.99	0.99	0.98	0.47	0.41
Site 2	0.99	0.98	0.98	0.97	0.97	0.81	1.42
Site 3	0.98	0.93	0.95	0.92	0.93	1.43	6.59
Site 4	0.98	0.91	0.94	0.90	0.93	1.87	8.41
Site 5	0.95	0.93	0.94	0.89	0.92	4.15	6.66
Site 6	0.99	0.95	0.97	0.95	0.96	0.33	4.38
Site 7	0.98	0.96	0.97	0.95	0.95	1.98	3.05
Site 8	0.98	0.98	0.98	0.96	0.96	1.18	1.85
Site 9	0.99	0.98	0.99	0.98	0.96	0.64	1.23
Site 10	0.98	0.96	0.97	0.94	0.95	1.54	3.58
Site 11	0.98	0.98	0.98	0.96	0.96	1.98	1.47
Site 12	0.99	0.96	0.98	0.96	0.65	0.06	3.80
Site 13	0.99	0.97	0.98	0.97	0.59	0.04	2.89
Site 14	0.98	0.92	0.95	0.91	0.91	1.24	7.12
Site 15	0.99	0.93	0.96	0.92	0.85	0.33	6.82
Site 16	0.99	0.93	0.96	0.93	0.72	0.28	6.56
Site 17	0.99	0.94	0.97	0.94	0.80	0.26	5.42
Site 18	0.99	0.96	0.98	0.96	0.16	0.00	3.67
Site 19	0.99	0.98	0.98	0.97	0.94	0.50	1.53
Site 20	1.00	0.99	0.99	0.99	N/A	0.00	0.98
**Macro-average**	**0.99**	**0.96**	**0.97**	**0.95**	**0.85**	**0.95**	**3.89**

**Table 6:** Point-wise FAST-GC performance under point-density reduction across the 20 benchmark sites. Values are site-level macro-averages. Balanced accuracy (BA), commission error (CE), omission error (OE). Original denotes the unchanged point cloud of the Jin et al. (2020) [17] benchmark; D6 (≈6 pts./m^2^) and D4 (≈4 pts./m^2^) denote the reduced-density datasets. Displayed values are truncated to two decimal places (not rounded).

Point Density	Precision	Recall	F1	IoU	MCC	CE	OE (%)	BA
Original	0.99	0.96	0.97	0.95	0.85	0.95	3.89	0.97
D6	0.98	0.96	0.97	0.95	0.85	1.15	3.28	0.97
D4	0.98	0.96	0.97	0.95	0.85	1.24	3.24	0.97

**Table 7:** FAST-GC DEM (0.5 m) accuracy under point-density reduction across the 20 benchmark sites. Values are macro-averages across the 20 benchmark sites. mean absolute error (MAE); root mean square error (RMSE); P95 absolute error, 95th percentile of absolute DEM error. Bias is FAST-GC DEM minus reference DEM. Displayed values are truncated to two decimal places (not rounded).

Point Density	Bias (m)	MAE (m)	RMSE (m)	P95 Absolute Error (m)
Original	−0.04	0.07	0.38	0.29
D6	−0.04	0.09	0.39	0.34
D4	−0.04	0.11	0.42	0.40

**Table 8:** Point-wise agreement of FAST-GC, PMF, CSF, and PTD with OpenGF for three mountainous scenes (S7_3, S8_6, and S9_14). Metrics include precision, recall, F1-score, intersection over union (IoU), Matthews correlation coefficient (MCC), commission error (CE), omission error (OE), and balanced accuracy (BA). CE and OE are relative to the supplied OpenGF reference labels. Bold values indicate the best performance for each metric.

Site	Algorithm	Precision	Recall	F1	IoU	MCC	CE (%)	OE (%)	BA
S7_3	FAST-GC	0.43	**0.98**	0.59	0.42	0.58	56.84	**1.87**	0.89
S7_3	PMF	**0.66**	0.89	**0.76**	**0.61**	**0.73**	**33.44**	10.78	**0.91**
S7_3	CSF	0.32	0.96	0.48	0.31	0.46	67.79	3.29	0.83
S7_3	PTD	0.58	0.94	0.72	0.57	0.70	41.09	5.15	**0.92**
S8_6	FAST-GC	0.73	**0.99**	0.84	0.72	0.70	26.94	**0.64**	0.84
S8_6	PMF	**0.78**	0.93	**0.85**	**0.74**	**0.71**	**21.90**	6.43	0.86
S8_6	CSF	0.71	0.99	0.83	0.71	0.68	28.61	0.68	0.83
S8_6	PTD	0.74	0.89	0.81	0.68	0.64	25.15	10.36	0.82
S9_14	FAST-GC	0.43	**0.94**	0.59	0.42	0.61	56.81	**5.44**	0.94
S9_14	PMF	**0.51**	0.84	**0.63**	**0.46**	**0.63**	**48.91**	15.58	0.90
S9_14	CSF	0.25	0.79	0.38	0.24	0.41	74.30	20.55	0.84
S9_14	PTD	0.45	0.93	0.61	0.44	**0.63**	54.37	6.07	0.94
Mean	FAST-GC	0.53	**0.97**	0.67	0.52	0.63	46.86	**2.65**	0.89
Mean	PMF	**0.65**	0.89	**0.75**	**0.60**	**0.69**	**34.75**	10.93	0.89
Mean	CSF	0.43	0.91	0.56	0.42	0.52	56.90	8.17	0.84
Mean	PTD	0.59	0.92	0.71	0.56	0.66	40.21	7.20	0.89

## Data Availability

Suggested Data Availability Statements are available in the section “MDPI Research Data Policies” at https://www.mdpi.com/ethics (accessed on 2 November 2026).

## Supplementary Materials

The following supporting information can be downloaded at: https://www.mdpi.com/article/10.3390/s26217032/s1, Figure S1: Stage-wise progression of ground classification F1-score across sequential FAST-GC processing stages in ULS mode; Supplementary Note S1: Sensor-Adaptive Configuration and Terrain-Classification Refinement in FAST-GC; Table S1: Sensor-specific core baseline configuration used to initialize FAST-GC; Table S2: Dataset-level automatic support-scale selection from median point count in 2 m grid cells; Table S2b: Point-density fallback used when the primary 2 m point-count statistic is unavailable; Table S3: Sensor-specific bounds applied to automatically adapted spatial-support parameters; Table S4: Fixed mathematical and structural controls used in the ALS/ULS Invert-Vote terrain classifier; Table S5: Production sequence of active classification and terrain-quality-control operations.
