# Supplementary material for: FAST-GC: A Fully Adaptive Self-Tuning Ground Classification Algorithm for Multi-Platform LiDAR Sensors
Source: Sensors (Basel). 2026 Nov 6;26(21):7032. doi: 10.3390/s26217032 (PMC13766393; doi:10.3390/s26217032)
Supplement: Supplementary file 1 [file sensors-26-07032-s001.zip › Note S1.pdf]

FAST-GC: A Fully Adaptive Self-Tuning Ground Classification Algorithm for Multi-Platform LiDAR Sensors

Supplementary Note S1 and Tables S1–S5

This supplementary document provides implementation-level details required to reproduce the sensor-adaptive terrain classification and refinement pathway used by FAST-GC. Symbols are kept consistent with the main manuscript: z_i denotes the original elevation of return i, z_i^I its elevation in inverted space, z_ref the common reference elevation, h_c the terrain-facing support elevation associated with horizontal cell c, T-hat the reconstructed terrain surface, r_i the point-to-terrain residual, s_i the local terrain-slope magnitude, tau_i the adaptive ground tolerance, and G_i the binary ground-state indicator.

Supplementary Note S1. Sensor-Adaptive Configuration and Terrain-Classification Refinement in FAST-GC

FAST-GC is designed so that the internal ground-classification parameters are not manually specified during standard processing. The user identifies the acquisition mode as airborne laser scanning (ALS), unmanned aerial laser scanning (ULS), or terrestrial laser scanning (TLS), after which FAST-GC initializes the corresponding sensor-specific baseline configuration. For all three sensor modes, selected spatial-support parameters are subsequently adapted automatically from dataset- and tile-level sampling statistics within predefined sensor-specific bounds. The values reported in Tables S1–S4 are therefore provided for algorithmic transparency and reproducibility rather than as parameters requiring dataset-specific manual optimization.

The adaptive configuration primarily modifies the spatial scale over which terrain evidence is collected while preserving the underlying terrain-first classification logic. ALS and ULS share the airborne Invert-Vote and terrain-quality-control pathway. TLS retains the same terrain-first principle but uses a dedicated inverted terrain-voting and classification pathway adapted to the anisotropic sampling geometry of terrestrial scanning.

S1.1. Dataset- and Tile-Level Adaptive Support

For each input dataset, FAST-GC derives a dataset-level support scale from the median point count of 2 m grid cells. Point density is used as a fallback when the primary 2 m support statistic is unavailable. The corresponding sensor-specific scale factors are reported in Table S2. A bounded tile-level adjustment is subsequently calculated from three relative sampling measures: the ratio between tile- and dataset-level median 2 m point counts, the difference in 2 m occupancy ratio, and the ratio between tile- and dataset-level point density.

$$\Delta s\_t \ = \ \Delta s\_(N,t) \ + \ \Delta s\_(O,t) \ + \ \Delta s\_(D,t) \quad \text{(S1)}$$

where Δs_t is the total tile-level scale adjustment, and Δs_(N,t), Δs_(O,t), and Δs_(D,t) are the contributions from relative 2 m point count, occupancy, and point density, respectively. The adjustment is constrained to the sensor-specific interval reported in Table S3 before combining the dataset-level scale.

The resulting effective scale s_eff controls the voting-cell size, candidate-cell size, base-cell size, and base support radius. The minimum number of supporting neighboring cells is adjusted inversely with the square root of the effective scale:

$$n\_min \ = \ clip[\, round(\, n\_min\hat{}(0) \ / \ \sqrt{s\_eff}\ ), n\_lo, n\_hi \,] \quad \text{(S2)}$$

where n_min^(0) is the sensor-specific baseline minimum support, and n_lo and n_hi are its lower and upper adaptive bounds. Thus, the spatial support expands under sparse sampling and contracts under denser sampling without requiring the user to specify a new classification configuration.

S1.2. Terrain-Void Recovery

Following initial ALS/ULS classification, FAST-GC evaluates locations in which observed low-elevation returns exist, but the classified terrain support is locally incomplete. Recovery is based on continuation from existing ground support rather than elevation alone. Directional terrain continuation is evaluated in the horizontal neighborhood. For candidate cell c, two already supported terrain cells lying along direction d provide a one-step terrain extrapolation:

$$\hat{z}\_(c,d) \ = \ 2\, z\_(c-d) \ - \ z\_(c-2d) \quad \text{(S3)}$$

The admissible residual increases with the local terrain step but remains bounded:

$$\tau\_(c,d) \ = \ min[\, \tau\_max, \tau\_b \ + \ k\_s\, |z\_(c-d) \ - \ z\_(c-2d)|\,] \quad \text{(S4)}$$

Directional support is accepted when

$$|z\_c \ - \ \hat{z}\_(c,d)| \ \le \ \tau\_(c,d) \quad \text{(S5)}$$

where τ_b is the baseline continuation tolerance, and τ_max is its upper bound. Recovery requires support from multiple directions, thereby reducing the probability that isolated low vegetation or detached returns are promoted solely because they occur below the reconstructed terrain membrane.

S1.3. Depression and Drainage Recovery

Terrain depressions can remain underrepresented when their elevations fall below terrain inferred from surrounding ground. FAST-GC therefore applies a dedicated depression-sweeping stage to ALS and ULS after initial void recovery. Observed low-return components are evaluated against surrounding classified ground and distinguished according to their spatial morphology. Compact or broad components are assessed as basin-like depressions, whereas elongated components are additionally evaluated using cross-sectional terrain support along their principal orientation. Validation considers depression depth, surrounding ground support, component width and depth variability, and, for elongated features, consistency among multiple cross sections.

$$G\_i\hat{}(p+1) \;=\; G\_i\hat{}(p) \,\vee\, D\_i\hat{}(p) \quad \text{(S6)}$$

where G_i^(p) is the current ground state of return i during pass p and D_i^(p)=1 only when return i belongs to a depression component satisfying the applicable geometric and terrain-support criteria. The operation is promotion-only and is limited to the bounded production pass sequence, preventing unrestricted recursive propagation.

S1.4. Terrain-Surface Consensus and False-Negative Recovery

After void and depression recovery, FAST-GC constructs a reusable spatial terrain-consensus representation from the observed point geometry. Candidate false-negative cells are identified from local point density, current ground occupancy, and low-return support. Existing classified ground surrounding each candidate is then used to estimate the local terrain manifold. For a local planar representation centered at (x_c, y_c),

$$\hat{z}(x,y) \;=\; a(x - x\_c) \;+\; b(y - y\_c) \;+\; c \quad \text{(S7)}$$

and the terrain-normal residual of return i is

$$d\_i \;=\; [\, z\_i \;-\; \hat{z}(x\_i, y\_i)\,] \,/\, \surd(1 \;+\; a^2 \;+\; b^2) \quad \text{(S8)}$$

where a and b are the local planar slopes, and c is the fitted elevation at the local coordinate origin. Where sufficient support is available, and local geometry requires it, the implementation can use a quadratic terrain representation rather than forcing curved terrain into a planar model. Recovery requires the candidate to remain within the sensor-specific terrain-normal tolerance and to receive sufficient spatial and directional support from neighboring terrain observations. The terrain-normal formulation reduces sensitivity to slope relative to a purely vertical-distance criterion.

S1.5. Bidirectional Near-Surface Classification Consistency

FAST-GC subsequently performs a bidirectional terrain-consistency test. Unlike promotion-only terrain recovery, this stage can promote a non-ground return or demote an existing ground return. A ground-labelled return can be demoted when it is sufficiently displaced above the locally supported terrain manifold, and its comparable-elevation neighborhood is dominated by non-ground observations. Conversely, a non-ground return can be promoted only when it lies sufficiently close to the local terrain manifold and receives strong neighboring ground support.

$$G\_i* = \{\, 0, if\ G\_i = 1, d\_i > \tau\_i\hat{}+, C\_i\hat{}NG \geq C\_min\hat{}NG \;;\;\; 1, if\ G\_i = 0, |d\_i| \leq \tau\_i\hat{}G, C\_i\hat{}G \geq C\_min\hat{}G \;;\;\; G\_i, otherwise \,\} \quad \text{(S9)}$$

where C_i^G and C_i^NG denote local terrain-consistent ground and non-ground evidence, respectively. The implementation additionally constrains these decisions using spatial support, component geometry, and terrain-manifold consistency.

S1.6. Final Airborne Terrain-Sheet Quality Control

The recovered ALS/ULS classification is subsequently subjected to a sequence of conservative terrain-sheet quality-control operations. These stages provide complementary tests rather than independent ground classifiers. The post-final surface stage targets remaining observed returns consistent with the reconstructed terrain sheet. This is followed by statistical ground-sheet consistency testing, three-dimensional facet support, ground-only vertical-profile analysis, detached-ground screening, surface-impurity screening, terrain-blob screening, vertical-support verification, and density-conditional final surface/context voting. The production order is summarized in Table S5.

These operations combine evidence including local terrain residuals, robust dispersion, point-density support, ground/non-ground neighborhood composition, local planar or quadratic surface agreement, vertical-profile modes, component morphology, and downward terrain connectivity. The quality-control operations modify classification labels only; they do not geometrically alter the original LiDAR coordinates. FAST-GC deliberately does not treat building or roof removal as a

universal core operation because a geometrically coherent elevated surface cannot always be removed without risking legitimate terrain. Anthropogenic-object cleanup can therefore be performed downstream when required by a specific application.

S1.7. TLS-Specific Terrain Pathway

TLS uses a dedicated terrain-voting implementation because terrestrial point clouds exhibit strongly anisotropic sampling, substantial vertical structure, occlusion, and highly variable local point density. The TLS pathway constructs locally coherent low-elevation terrain candidates, incorporates offset-grid support, evaluates spatial support and confidence, and applies a terrain-adaptive point-classification rule. Its vertical residual retains the same sign convention as the main manuscript:

$$r\_i\hat{}TLS = z\_i - T\_TLS(x\_i, y\_i) \quad \text{(S10)}$$

where T̂_TLS(x_i,y_i) is the TLS terrain surface sampled at return i. The TLS implementation uses dedicated internal controls for local support, roughness, vertical structure, and terrain continuity rather than applying the ALS/ULS refinement sequence unchanged.

In the production repository evaluated for this manuscript, the modules tls_membrane_refine.py and tls_terrain_recover.py are retained as compatibility interfaces but are implemented as no-operation shims: the candidate ground mask is returned unchanged, and no additional post-membrane microtopographic recovery is applied. Consequently, these compatibility modules are not treated as active classification stages in Table S5.

S1.8. Buffered-Tile Processing and Merge

When tiled processing is used, classification is performed using both the target tile and its surrounding spatial buffer. The buffer supplies neighborhood evidence to terrain estimation and classification near tile boundaries. During merging, only observations inside each predefined core tile extent are retained. Buffer observations therefore contribute contextual support without being duplicated in the final merged point cloud. Raster products are likewise cropped to the corresponding core extent before mosaicking.

S1.9. Interpretation of Self-Tuning Operation

FAST-GC does not require manual specification or dataset-specific tuning of its internal ground-classification parameters during standard operation. The user specifies the acquisition mode (ALS, ULS, or TLS), after which the corresponding internal baseline configuration is initialized automatically. Selected spatial-support parameters are then adjusted from observed dataset- and tile-level sampling statistics within predefined sensor-specific bounds. Tables S1–S4 document these internal values for transparency and reproducibility; they are not required user inputs.

Table S1. Sensor-specific core baseline configuration used to initialize FAST-GC.

Internal parameter	Symbol	ALS	ULS	TLS	Unit
Voting-cell size	h_0	1.00	0.75	0.35	m
Candidate top support	—	3	4	12	count
Voting-neighborhood radius	R_0	3	4	4	cells
Minimum neighboring support	$n\_min^{(0)}$	6	8	8	cells
Robust-deviation multiplier	k_MAD	3.0	2.8	2.6	—
MAD floor	d_min	0.05	0.05	0.03	m
Maximum filling iterations	—	80	100	24	iterations
Surface smoothing	σ	1.20	1.00	0.65	cells
Baseline ground tolerance	τ_0	0.30	0.18	0.10	m
Slope-adaptation coefficient	k_s	0.40	0.30	0.22	—
Candidate-cell size	—	2.00	1.50	2.00	m
Candidate vertical band	—	0.75	0.60	0.20	m
Base-cell size	—	2.00	1.50	2.00	m
Base support radius	—	1.00	0.50	0.25	m
Generic void recovery	—	Enabled	Enabled	Disabled	—

Note: Values are internal sensor-specific initialization values implemented in the production configuration and are not user-tuned parameters. Selected spatial quantities are subsequently adapted when dataset- or tile-level support statistics are available. TLS additionally uses dedicated internal terrain-voting controls.

Table S2. Dataset-level automatic support-scale selection from median point count in 2 m grid cells.

Sensor	Median point count per 2 m cell	Automatic scale, s
ALS	<10	1.60
ALS	10–<25	1.40
ALS	25–<60	1.20
ALS	60–<160	1.00
ALS	160–<350	0.90
ALS	≥350	0.82
ULS	<20	1.45
ULS	20–<50	1.25
ULS	50–<120	1.10
ULS	120–<300	1.00

ULS	300–<700	0.92
ULS	≥700	0.86
TLS	<40	1.50
TLS	40–<100	1.30
TLS	100–<250	1.15
TLS	250–<700	1.00
TLS	700–<1800	0.92
TLS	≥1800	0.86

Note: These scale bands are algorithm-defined, sensor-specific adaptive rules. They are not constants taken from literature. The median 2 m cell point-count statistic is the primary dataset-level scale selector.

Table S2b. Point-density fallback used when the primary 2 m point-count statistic is unavailable.

Sensor	Point density (points m^{-2})	Automatic scale, s
ALS	<5; 5–<15; 15–<40; 40–<120; 120–<250; ≥250	1.60; 1.35; 1.15; 1.00; 0.90; 0.82
ULS	<10; 10–<30; 30–<90; 90–<220; 220–<500; ≥500	1.40; 1.22; 1.10; 1.00; 0.92; 0.86
TLS	<25; 25–<80; 80–<200; 200–<600; 600–<1400; ≥1400	1.45; 1.25; 1.12; 1.00; 0.92; 0.86

Note: Point density is used only as a fallback dataset-scale statistic when the primary 2 m point-count statistic is unavailable.

Table S3. Sensor-specific bounds applied to automatically adapted spatial-support parameters.

Adaptive quantity	ALS	ULS	TLS	Unit
Effective scale, s_eff	0.80–1.80	0.80–1.60	0.80–1.55	—
Voting cell	0.75–2.00	0.50–1.50	0.25–0.80	m
Candidate cell	1.50–4.00	1.00–3.50	1.00–3.00	m
Base cell	1.50–4.00	1.00–3.50	1.00–3.00	m
Base radius	0.75–2.50	0.35–1.50	0.15–0.90	m
Minimum neighboring cells	4–8	6–10	8–14	cells
Tile-scale adjustment	−0.10–+0.12	−0.08–+0.10	−0.08–+0.10	—

Note: Tile-level adjustment rules: relative to the dataset median, a tile point-count ratio <0.50 contributes +0.10 to the scale adjustment and 0.50–<0.80 contributes +0.05; ratios >1.80 and >2.50 contribute −0.05 and −0.08, respectively. An occupancy difference below −0.10 or −0.20 contributes +0.025 or +0.05, whereas differences above +0.10 or +0.20 contribute −0.015 or −0.03. A tile-to-dataset density ratio <0.50 contributes +0.04, whereas a ratio >2.00 contributes −0.03. The summed adjustment is clipped to the sensor-specific interval shown above.

Table S4. Fixed mathematical and structural controls used in the ALS/ULS Invert-Vote terrain classifier.

Control	Value	Interpretation
Offset-grid displacement	0.5h	Structural algorithm constantly reducing grid-origin sensitivity
MAD consistency factor	1.4826	Gaussian-consistent scaling of the median absolute deviation
Median-fill neighborhood	3 × 3	Structural neighborhood for unsupported voted-terrain cells

Breakline-relief multiplier	1.5	Internal multiplier for local terrain-transition allowance
Maximum breakline allowance	0.25 m	Upper bound on the transition contribution
Minimum adaptive ground tolerance	0.05 m	Lower bound on terrain-adaptive classification tolerance
Maximum adaptive ground tolerance	0.65 m	Upper bound on terrain-adaptive classification tolerance
Lower residual multiplier	2.0	Defines the asymmetric lower residual interval

Note: The factor 1.4826 is the conventional Gaussian-consistent MAD scaling factor. The remaining values are structural or implementation constants/bounds of the production algorithm and should not be interpreted as externally prescribed literature parameters.

Table S5. Production sequence of active classification and terrain-quality-control operations.

Seq.	Operation	ALS	ULS	TLS	Primary effect	Implementation
1	Initial Invert-Vote terrain classification	✓	✓	—	Initial G/NG	invert_vote.py
1	Dedicated TLS terrain vote/classification	—	—	✓	Initial G/NG	tls_vote.py
2	Terrain-void recovery	✓	✓	—	Promotion	void_recover.py
3	Depression/ditch recovery	✓	✓	—	Promotion	depression_sweeper.py
4	Surface false-negative recovery	✓	✓	—	Promotion	surface_consensus_guard.py
5	Two-way surface consistency	✓	✓	—	Promotion + demotion	surface_consensus_guard.py
6	Post-final terrain completion	✓	✓	—	Promotion	als_postfinal_surface.py
7	Statistical ground-sheet cleaner	✓	✓	—	Demotion	als_statistical_sheet_cleaner.py
8	3-D facet-support consistency	✓	✓	—	Demotion	als_facet_support_guard.py
9	Ground vertical-profile guard	✓	✓	—	Demotion	als_ground_vertical_profile_guard.py
10	Detached-ground guard	✓	✓	—	Demotion	als_detached_ground_guard.py
11	Surface-impurity guard	✓	✓	—	Demotion	als_surface_impurity_guard.py
12	Terrain-blob/canopy-impostor guard	✓	✓	—	Demotion	als_terrain_blob_guard.py
13	Vertical-support final guard	✓	✓	—	Demotion/QC	vertical_support_final_guard.py
14	Final normal-space surface vote	Density-conditional	Density-conditional	—	Demotion	als_final_surface_vote.py

15	D2 reverse multiscale context vote	Very-low-density conditional	Very-low-density conditional	—	Demotion	als_d2_reverse_context_vote.py
16	Post-reverse final-surface polish	Very-low-density conditional	Very-low-density conditional	—	Demotion	als_final_surface_vote.py

Note: G/NG = ground/non-ground. The table reports the production call sequence represented in the manuscript release. Some late airborne guards are activated conditionally by the internally determined sampling-density regime; they are therefore listed as density-conditional rather than as separate user-selected processing modes. The retained TLS membrane-refinement and microtopographic-recovery interfaces are compatibility no-operation shims in this production baseline and are not listed as active stages.

Supplementary Nomenclature

Symbol	Definition
i	Point/return index
c	Horizontal grid-cell index
d	Horizontal continuation direction
z_i	Original elevation of return i
z_i^I	Elevation of return i in vertically inverted space
z_{ref}	Common reference elevation used for vertical inversion
h_c	Terrain-facing support elevation associated with cell c in the main Invert-Vote formulation
$\hat{T}(x,y)$	Reconstructed terrain surface sampled at horizontal location (x,y)
r_i	Vertical point-to-terrain residual, $z_i - \hat{T}(x_i,y_i)$
s_i	Local terrain-slope/gradient magnitude
τ_i	Terrain-adaptive classification tolerance
G_i	Binary ground-state indicator for return i
s_{eff}	Effective sensor- and sampling-adaptive spatial-support scale
n_{min}	Minimum number of supporting neighboring cells
d_i	Terrain-normal residual used by local terrain-consensus refinement
C_i^G, C_i^{NG}	Local terrain-consistent ground and non-ground evidence

Note: Symbols already used in the main manuscript retain the same definitions and sign conventions. New symbols introduced only for supplementary refinement equations are defined locally and in this nomenclature.
